# Low-intensity ultrasound restores long-term potentiation and memory in senescent mice through pleiotropic mechanisms including NMDAR signaling

**DOI:** 10.1038/s41380-021-01129-7

**Published:** 2021-05-27

**Authors:** Daniel G. Blackmore, Fabrice Turpin, Tishila Palliyaguru, Harrison T. Evans, Antony Chicoteau, Wendy Lee, Matthew Pelekanos, Nghia Nguyen, Jae Song, Robert K. P. Sullivan, Pankaj Sah, Perry F. Bartlett, Jürgen Götz

**Affiliations:** 1 Clem Jones Centre for Ageing Dementia Research, Queensland Brain Institute, The University of Queensland, Brisbane, QLD Australia; 2 Queensland Brain Institute, The University of Queensland, Brisbane, QLD Australia; 3 Joint Center for Neuroscience and Neural Engineering, and Department of Biology, Southern University of Science and Technology, Shenzhen, Guangdong Province P. R. China

**Keywords:** Biochemistry, Neuroscience

## Abstract

Advanced physiological aging is associated with impaired cognitive performance and the inability to induce long-term potentiation (LTP), an electrophysiological correlate of memory. Here, we demonstrate in the physiologically aged, senescent mouse brain that scanning ultrasound combined with microbubbles (SUS^+MB^), by transiently opening the blood–brain barrier, fully restores LTP induction in the dentate gyrus of the hippocampus. Intriguingly, SUS treatment without microbubbles (SUS^only^), i.e., without the uptake of blood-borne factors, proved even more effective, not only restoring LTP, but also ameliorating the spatial learning deficits of the aged mice. This functional improvement is accompanied by an altered milieu of the aged hippocampus, including a lower density of perineuronal nets, increased neurogenesis, and synaptic signaling, which collectively results in improved spatial learning. We therefore conclude that therapeutic ultrasound is a non-invasive, pleiotropic modality that may enhance cognition in elderly humans.

## Introduction

**Fig. 1:**
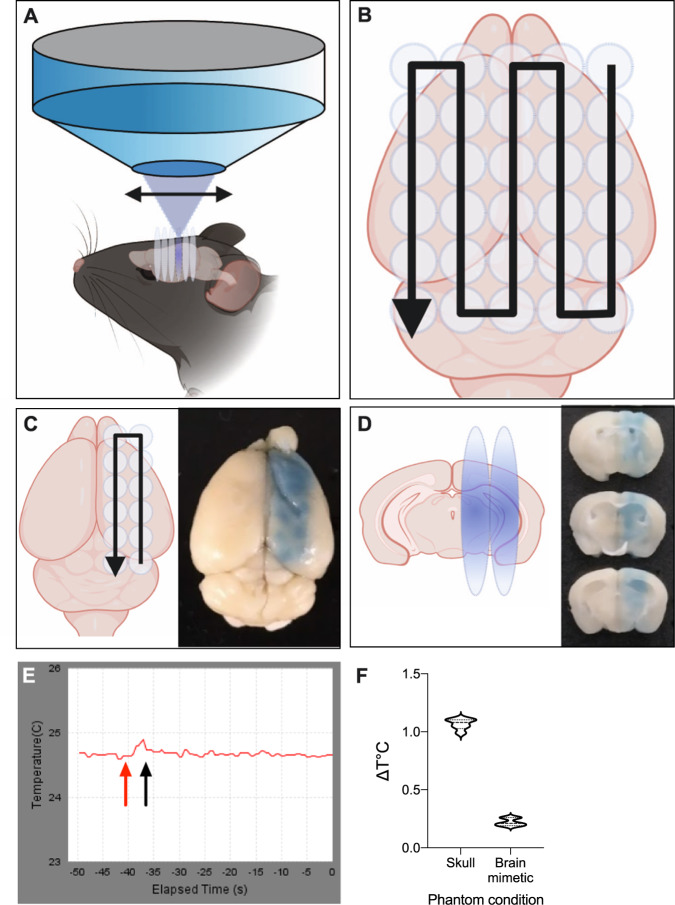
The scanning ultrasound equipment accurately delivers treatment and does not generate aversive levels of heat. **A** Schematic detail of the sonication procedure, indicating that the transducer sonicates a large area of the mouse brain during each treatment. **B** Schematic of the movement pattern of the transducer (black arrow). The start and end points are specified, and the transducer was scanned over the area in a raster grid pattern. A typical whole brain sonication used a 5 × 6 spot sonication pattern, covering virtually all of the cerebrum. Each mouse was rotated 180° for subsequent treatments. **C** To highlight the specificity of the sonication process, less than half of the brain was scanned using a 2 × 6 spot sonication path. The illustration shows a mouse brain following such a SUS^+MB^ sonication to regionally open the BBB, with Evans blue extravasation visualizing this scanning pattern. **D** The brain from (**C**) cut coronally in 1 mm sections, with the Evans blue staining showing that ultrasound penetrated through the entirety of the brain. **E** Representative temperature versus time plot for the sonication using a gray matter mimetic. Note the temperature rise beginning at ~40 s of the elapsed time (red arrow), and the rapid decline after the 6 s sonication ended (black arrow). **F** Average temperature change (ΔT °C) measured at the skull and with gray matter mimetic 6 s after sonication (*n* = 6, mean ± SD).

Physiological aging leads to a progressive decline in the functional and cellular constituents of the brain [1]. Whether the age-dependent decline in memory functions can be slowed or even reversed remains to be determined. Here, we explored the neuromodulatory potential of low-frequency therapeutic ultrasound in aged, cognitively impaired C57BL/6 wild-type (WT) mice, building on earlier work that not only demonstrated the long-term safety of this technology [2] (Fig. 1A), but also that pathological changes and the ensuing cognitive deficits can be ameliorated in Alzheimer’s disease (AD) mouse models [3, 4]. In these earlier studies, focused ultrasound was delivered through the skull into the brain in a scanning ultrasound (SUS) mode in animals that had received an intravenous injection of microbubbles (MBs: SUS^+MB^). As the MBs undergo repetitive cycles of compression and rarefaction in response to ultrasound, the tight junctions of the endothelial cells lining the blood vessels become disrupted, transiently opening the blood–brain barrier (BBB) [5]. This, together with the activation of vesicular, trans-cytoplasmic transport [6], allows for the uptake of as yet unidentified blood-borne factors into the brain, which elicit a wide range of therapeutic effects, including the activation of microglia to take up and remove protein aggregates [3].

In the current study we asked whether SUS represents a cognition enhancement tool under conditions of physiological aging, and whether this requires BBB opening. Thus, we chose to evaluate therapeutic ultrasound with or without BBB opening (SUS^+MB^ versus SUS^only^) in physiologically aged, senescent mice. With a maximal lifespan of approximately 26 months [7], 12-month-old WT mice are still capable of spatial learning as revealed by the active place avoidance (APA) paradigm [2]. However, cognitive function subsequently deteriorates [8] and, by 18 months, spatial learning is severely compromised [9]. Here, we used 20- to 22-month-old mice to determine whether therapeutic ultrasound can restore cognitive functions in the aged mouse brain.

## Materials and methods

### Animal ethics

Animal experimentation was approved by the Animal Ethics Committee of the University of Queensland (approval numbers QBI/412/14/NHMRC, QBI/348/17/NHMRC, and QBI/554/17/NHMRC). The animals were housed in specific pathogen-free cages and maintained on a 12-h light/dark cycle, with unlimited access to food and water.

### Study design and statistical analysis

All mice used in the study were female C57Bl/6 WT mice. Following an earlier protocol [2], we performed SUS with and without MBs (SUS^+MB^ and SUS^only^) on the WT mice (20 months old at start and 22 months old at completion), using an age-matched sham group (anesthetized, head restrained but no MBs or SUS treatment) as control (Supplementary Fig. S1). In the SUS mode, a motorized positioning system moves the focus of the transducer array in a grid such that the ultrasound is delivered sequentially to the entire brain [3, 4]. The animals all underwent a weekly treatment for six weeks prior to behavioral testing, using an accelerated APA paradigm. *N* values for each treatment were: *N* = 18 (sham), *N* = 15 (SUS^+MB^), *N* = 20 (SUS^only^). Mice were sacrificed two days after the APA test. Naive mice (*N* = 6) aged 22 months were included as an additional control to rule out the potential confound of anesthesia.

Researchers were blinded to phenotype during experimentation. Following APA testing, a subset of mice was used to obtain slice cultures for electrophysiological recordings to measure induction of long-term potentiation (LTP): *N* = 3 (sham), *N* = 4 (SUS^+MB^), and *N* = 3 (SUS^only^), using two slices per animal. The remainder of the mice were perfused, and their brains dissected for histological or biochemical analysis. For the latter, *N* = 7 (sham), *N* = 8 (SUS^+MB^), and *N* = 11 (SUS^only^) were used, assessing total protein (TP) and the postsynaptic density (PSD) fraction. For sequential window acquisition of all theoretical fragment ions mass spectrometry (SWATH-MS), total hippocampal protein extracts were used from *N* = 5 (sham), *N* = 5 (SUS^+MB^), and *N* = 5 (SUS^only^) mice. For immunohistochemistry, paraffin sections were obtained from *N* = 5 (naive), *N* = 7 (sham), *N* = 7 (SUS^+MB^), and *N* = 9 (SUS^only^) mice (Supplementary Fig. S1).

Factorial design one-way analysis of variance (ANOVA) with Bonferroni’s multiple comparison post hoc test, Student’s *t*-test, or correlation analysis was used to analyze data, as appropriate. Parameters were analyzed in Microsoft Excel and Prism 8 (GraphPad Software Inc.). All values were expressed as mean ± standard error of the mean, unless otherwise indicated. Comprehensive statistical descriptions and results for every figure and supplementary figure are found in Supplementary Table S1. Sample sizes for power calculations were conducted using the Power/Sample size calculator website generated by the University of British Columbia (Canada): https://www.stat.ubc.ca/~rollin/stasts/ssize/. Sample sizes were based on the mean of populations 1 and 2, common standard deviation (sigma value), an alpha value setting of 0.05, and a desired power value of 0.80.

### SUS equipment

An integrated focused ultrasound system was used (Therapy Imaging Probe System, TIPS, Philips Research) [10] (Supplementary Fig. S2A). The system consisted of an annular array transducer with a natural focus of 80 mm, a radius of curvature of 80 mm, a spherical shell of 80 mm with a central opening of 31 mm diameter, a 3D positioning system, and a programmable motorized system to move the ultrasound focus in the *x* and *y* planes to cover the entire brain area. A coupler mounted to the transducer was filled with degassed water and placed on the depilated head of the mouse with ultrasound gel for coupling, to ensure propagation of the ultrasound to the brain. The focal zone of the array was an ellipse of approximately 1.5 mm × 1.5 mm × 12 mm.

### Production of microbubbles

Lipid-shelled MBs with an octafluoropropane core were manufactured and characterized in-house. A 1:5:2:1 mass ratio of PEG6000, distearoyl-phosphatidylcholine, distearoylphosphatidylethanolamine, and pluronic F68 was dissolved in a 0.9 % solution of sodium chloride. The solution was added to glass HPLC vials and the air was removed and replaced with octafluoropropane gas to fill the headspace of the vial (Arcadophta). On the day of use, vials were heated to 37 °C and then shaken in a dental amalgamator for 40 s at 4,000 rpm. The concentration and size of the MBs was examined under a microscope and found to be 1–5 × 10^7^ MBs/ml with a size range of 1–10 μm, and a mean diameter of 4 μm, using a Coulter counter.

### SUS application

Mice were anesthetized with ketamine (100 mg/kg) and xylazine (10 mg/kg) and the hair on the head was shaved and depilated. For the SUS^+MB^ group, mice were injected retro-orbitally with 1 µl/g body weight of MB solution and then placed under the ultrasound transducer with the head immobilized. SUS^only^ animals were similarly anesthetized but did not receive MB injections. The delay between MB injection and SUS onset was approximately 30 s. The parameters for ultrasound delivery were: 0.7 MPa peak rarefactional pressure, 10 Hz pulse repetition frequency, 10 % duty cycle, and a 6 s sonication time per spot. The motorized positioning system moved the focus of the transducer array in a grid with 1.5 mm between individual sites of sonication so that ultrasound was delivered sequentially to the entire brain (Fig. 1A, B). For sham treatments, mice received all injections and were placed under the ultrasound transducer, but no ultrasound was emitted. Animals were ear notched and researchers were blinded to treatment. To confirm that BBB opening was only achieved after SUS^+MB^ treatments, mice in three groups (*N* = 2 for each: naive, SUS^+MB^, and SUS^only^) were injected with 30 μl Evans blue dye (E2129, Sigma, 2% in saline) immediately after sonication. The mice were sacrificed by transcardial perfusion 60 min after sonication, after which their brains were fixed in 4% paraformaldehyde (PFA) overnight and imaged at 700 nm for 30 s in a fluorescence scanner (Odyssey Fc, LI-COR).

### Measurement of heat during SUS application

The sonication parameters used in this study were of low energy (with a peak acoustic power of 0.12 W) and heating was not considered a defining factor. To confirm this, numerical estimates (Supplementary Text) and phantom experiments were conducted (Fig. 1 and Supplementary Fig. S2B). A simple heat measurement apparatus was constructed using the TIPS system’s temperature measurement facilities and a gray matter mimetic (acoustic phantom). The TIPS system is capable of online heat measurement for thermal ablation studies, and it came equipped with T-type bare-wire thermocouples approximately 100 μm thick. Tests were performed by measuring the temperature change (ΔT, in °C) after sonication, using the TIPS system’s “GetDeltaTemperature” event command. This command automatically records the temperature before and after sonication and provides a readout of the temperature difference.

A sonication phantom was constructed from a 10 mm × 10 mm × 5 mm block of gray matter mimetic gel (True Phantom Solutions, Windsor, Canada), into which a thermocouple was inserted flush with the top face. The mimetic was positioned in place of a mouse under the TIPS transducer, placed on a stack of acoustic absorbent rubber and coupled to the transducer with coupling gel. The sonication was targeted directly onto the thermocouple. In a second setup, a piece of fixed mouse skull was placed on top of the thermocouple and sonicated as above (Supplementary Fig. S2B).

Using this apparatus, the TIPS system’s “Scout Temperature” function was employed to target the transducer focus onto the thermocouple. Briefly, this function scans the transducer over a defined area during sonication, and plots the temperature rise in each spot. The spot with the greatest temperature rise was then chosen for the remainder of the experiment. The ΔT in °C was reliably detected after 6 s (Fig. 1E), which is the duration of each sonication spot in the mouse treatments throughout this study. The apparatus was allowed to equilibrate to an ambient temperature for 20 min before sonication, and five sonications for each condition (with or without skull) were performed. Between sonications, the gray matter mimetic was allowed to cool to baseline temperature using the scrolling temperature reading.

Another readout as a proxy for heat induction was a proteomic examination, by determining changes in heat-shock proteins (see below).

### Active place avoidance (APA) test

The APA task is a test of hippocampus-dependent spatial learning [3]. The mice were tested over 5 days in a rotating elevated arena (Bio-Signal group) that had a grid floor and a 32 cm high clear plastic circular fence enclosing a total diameter of grid of 77 cm. High-contrast visual cues were present on the walls of the testing room. The arena and floor rotated at a speed of 1 rpm and a 500 ms, 60 Hz, 0.5 mA mild shock was delivered through the grid floor when the animal entered a 60° shock zone, and every 1,500 ms until it left this zone. The shock zone was maintained at a constant position in relation to the room. Recorded tracks were analyzed with Track Analysis software (Bio-Signal group). A habituation session was performed 24 h before the first training session in which the mice were placed in the rotating arena for 5 min to explore but did not receive any shocks during this period. The following day, animals were tested in a single 30 min training session. The number of shocks delivered, and the maximal time spent avoiding the aversive shock zone were compared between the sham, SUS^+MB^, and SUS^only^ treatment groups. To examine learning ability during the task, results were binned into 5 min intervals. Learning ability was calculated by comparing the performance in the first 5 min of the task to the last 5 min of the task and represented as a percentage. Data were analyzed using one-way ANOVA with Bonferroni post hoc analysis.

### Processing of brain tissue and subcellular fractionation

Following transcardial perfusion of the mice with ice-cold PBS, one half of the brain was fixed in 4% PFA for 24 h at 4 °C before being processed for immunohistochemical analysis. The other brain hemisphere was used to dissect the hippocampus, cortex, and cerebellum, with tissue placed in Eppendorf tubes and snap frozen in dry ice prior to storage at −80 °C until required.

Subcellular fractionation was performed on hippocampi with slight modifications of a previously described method [11]. Frozen brain tissue was first homogenized on ice in sucrose buffer (0.32 M sucrose, 10 mM HEPES, pH 7.4). An aliquot of homogenate was sonicated and used as the TP fraction. The remaining homogenate was centrifuged at 1,000 g for 10 min at 4 °C, yielding a supernatant fraction (S1) and a nuclear enriched pellet (P1). The P1 pellet was resuspended in RIPA buffer, followed by sonication to produce a nuclear fraction (N). The supernatant (S1) was centrifuged at 14,000 g for 20 min at 4 °C to obtain a crude synaptosomal fraction (P2) and a cytosolic protein (Cyto) enriched supernatant (S2). The P2 pellet was washed twice with wash buffer (4 mM HEPES, 1 mM EDTA, pH 7.4) followed by resuspension and centrifugation at 12,000 g for 20 min at 4 °C. It was then resuspended in buffer A (20 mM HEPES, 100 mM NaCl, 0.5% Triton X‐100, pH 7.2). After rotation at 4 °C for 1 h, the suspension was centrifuged at 12,000 g for 20 min at 4 °C to yield the supernatant S3 non‐PSD fraction containing extrasynaptic proteins. The resulting pellet (P3) was washed twice in wash buffer and resuspended in buffer B (20 mM HEPES, 0.15 mM NaCl, 1% Triton X‐100, 1% SDS, 1 mM dithiothreitol, 1% deoxycholate, pH 7.5) for 1 h at 4 °C, followed by centrifugation at 10,000 g for 20 min at 4 °C to obtain the supernatant S4 PSD fraction containing synaptic proteins (PSD). All buffers were freshly supplemented with protease and phosphatase inhibitor cocktail (Merck/Sigma) prior to use, and fractions were stored as aliquots at −80 °C.

### SWATH mass spectrometry

To identify changes in protein abundance following SUS^only^ and SUS^+MB^ treatment compared to sham, total hippocampal protein extracts from five mice per group were analyzed by SWATH-MS. Briefly, 45 µg of protein from each sample was diluted in triethylammonium bicarbonate buffer and subsequently reduced with DTT, followed by alkylation with iodoacetamide. Samples were then digested with 80 ng of trypsin overnight, after which they underwent a buffer exchange to be resuspended in 2% acetonitrile, 0.1% formic acid. In order to form a unique peptide library, 5 µg from each sample was first pooled together and then fractionated via high pH reversed-phase high-performance liquid chromatography to form 18 fractions that were then analyzed via data-dependent acquisition (IDA). This, in addition to SWATH-MS analysis, was performed as previously described, with significantly regulated proteins being defined as *p* value ≤ 0.05 (*t*-test) and a fold-change ≥ ±1.5 [12].

### Bioinformatics analysis of SWATH-MS data

Network and clustering analyses were performed using Cytoscape (v3.6.0) and MCODE. Data from the SWATH-MS analysis were mapped to the STRING protein query database for *Mus musculus* using the UniProt identifier. A confidence of interaction score cut-off of 0.15 was used. Network maps of the proteins that exhibited significantly altered abundance between groups (*p* ≤ 0.05, FC ≥ ±1.5) were then generated using the Edge-weighted Spring embedded Layout. Gene ontology (GO) analysis was performed using the ShinyGO (v0.61) [13].

### Western blotting

Equal amounts of protein were loaded (10–20 μg depending on the primary antibody used) and resolved by SDS–PAGE (4–15% Criterion TGX Precast Gels from Bio-Rad) in Tris‐glycine‐SDS buffer (Bio-Rad), followed by transfer onto low fluorescence polyvinylidene fluoride membranes (Bio‐Rad) as previously described [11]. After transfer, the membranes were incubated with REVERT total protein stain (LI-COR) for 5 min and washed in wash buffer (6.7% v/v glacial acetic acid, 30% v/v methanol in water), then scanned with the 700 nm channel in the Odyssey Fc Imaging system (LI-COR) for loading control analysis. The membranes were incubated in REVERT reversal solution (0.1 M sodium hydroxide, 30% v/v methanol in water) for 5 min. They were then blocked with Odyssey blocking buffer (LI-COR) for 1 h, and reacted with primary antibodies in Odyssey blocking buffer (LI-COR) in Tris‐buffered saline containing 0.05% Tween‐20 (TBS‐T) overnight at 4 °C under gentle rocking. Primary antibodies are listed (Table 1). After washing in TBS-T, secondary antibodies conjugated with IR 680RD/800CW (LI-COR 1:10,000) were added for 1 h at room temperature under gentle rocking. After washing in TBS-T, the membranes were scanned in the Odyssey Fc Imaging system (LI-COR) for detection of an infrared signal. A corresponding identical control sample for each fraction was loaded onto every gel to normalize signal differences from gel to gel. Where several antibodies were employed, the blots were cut into stripes before probing; however, to confirm the specificity and lack of background staining, representative full immunoblots are provided for brain lysates for antibodies frequently used throughout this study.

**Table 1:** Antibodies used.

Reagent or resource	Analysis	Dilution	Company	Cat. no.
NR2a	WB	1:1000	Merck	AB10531
NR2b	WB	1:500	NeuroMab UC Davis	75-097
Phospho-NR2b phospho-Y-1472	WB	1:500	Sigma	M2442
ERK	WB	1:1000	Cell Signaling Technology	#4696
Phospho-ERK 1/2	WB	1:1000	Cell Signaling Technology	4370
S6	WB	1:1000	Cell Signaling Technology	#2317
Phospho-S6	WB	1:1000	Cell Signaling Technology	#4858
PSD95	WB	1:1000	Merck Millipore	MABN68
Actin	WB	1:2000	Merck Millipore	MAB1501R
Glt-1 (EAAT1)	IHC	1:1000	ThermoFisher	PA5-17099
TRPA1	WB	1:500	Merck Millipore	ABN1009
GAP43	WB	1:1000	Merck Millipore	MAB347
Serine racemase (D5V9Z)	WB	1:500	Cell Signaling Technology	#29798
GLAST (EAAT2)	IHC	1:1000	ThermoFisher	PA5-19709
GFAP	IHC	1:2000	Synaptic Systems	173004
DCX	IHC	1:500	Cell Signaling Technology	4604S
WFA-biotin	IHC	1:2500	Vector Labs	B1355
HABP-biotin	IHC	1:400	AMSBIO	AMS.HKD-BC41
GFAP	IHC	1:1000	Abcam	ab4674
Iba1	IHC	1:1000	Wako Japan	#019-19741
Goat anti-chicken-488	IHC	1:1000	Invitrogen	A-11039
Goat anti-rabbit-647	IHC	1:1000	Invitrogen	A-32733
Anti-mouse IRDye800CW	WB	1:10,000	LiCor	926-32210
Anti-rabbit IRDye680RD	WB	1:10,000	LiCor	926-68070

### Immunohistochemistry

Upon sacrifice, animals underwent transcardiac perfusion with PBS only. Brains were removed and segregated sagittally into two halves. One half was drop fixed in 4% PFA for 24 h at 4 °C before being processed for sectioning and immunohistochemical analysis. The other half was dissected in ice-cold PBS with the cortex, hippocampus, and cerebellum collected and immediately snap frozen in dry ice before being stored at −80 °C until required for sample preparation and western blot analysis. The remaining animals were transcardially perfused with PBS followed by 4% PFA within 2 h of completing behavioral testing. Brains were cut in half in a sagittal direction with half post-fixed in 4% PFA, whereas the remaining half was drop fixed in glutaraldehyde .

### Image analysis of histological sections

Tissue sections (4 µm thick) were paraffin-embedded onto microscopy slides. Slides were baked for 30 min and dewaxed using xylene and ethanol (100–70%). They were then immersed in antigen retrieval solution in a pressure cooker at 60 °C for 30 min. Sections were blocked using 0.5% bovine serum albumin, 0.05% saponin, and 0.05% sodium azide in PBS. The following primary antibodies (Table 1) were used: rabbit anti-doublecortin (DCX 1:500, Cell Signaling Technology) and biotin-Wisteria floribunda agglutinin (WFA, 1:2,500, Vector Labs). Secondary antibodies were donkey anti-rabbit 488 (1:1,000, Abcam), and streptavidin 555 (1:1,000, Molecular Probes) plus 4′,6-diamidino-2-phenylindole (DAPI, 1:5,000, Sigma-Aldrich). Imaging for fluorescence was performed using a Yokogawa W1 spinning disk confocal microscope, and Axio Imager Green. For quantitative analysis, discrete counts of DCX-expressing immature neurons and WFA-labeled perineuronal nets (PNNs) were performed in the dentate gyrus of the hippocampus of these sections under visual inspection.

Images were captured using Zen software for Carl Zeiss light microscopy imaging. Axio Fluorescent images were captured under 10× magnification at 488 nm (DCX) and 555 nm (WFA) for 300 and 250 ms, respectively, with DAPI at 10 ms. Confocal images were taken under a 63× objective (oil immersion) for 100 ms (A488 channel), 55 ms (A568 channel), and 20 ms (DAPI Quad) under oil immersion. Fiji software was used for assemblage of high objective (63× with oil immersion) *z*-stacked images for elucidation of DCX^+ve^ and WFA^+ve^ cellular processes −0.27 µm Z step size. Z stacks in 16-bit TIFF format were z-projected in FIJI, using the average intensity to flatten the images, and were designated a color based on the channel wavelength.

### Astrocyte and microglial analysis

Immunofluorescence staining for microglia (Iba1) and astrocytes (GFAP) was performed over 3 days, using the reagents described above. On the first day, heat-fixed, paraffin-embedded mouse tissue slides were dewaxed, rehydrated, and antigen retrieved at 60 °C for 30 min, and then blocked for 1 h at room temperature. Primary antibody solution contained 1:1000 rabbit anti-Iba1 (Wako Japan #019-19741) and 1:1000 chicken anti-GFAP antibodies in blocking solution. Blocking solution with no primary antibodies was used as negative control. After overnight incubation, the slides were rinsed three times in PBS and then blocked for 10 min before application of secondary antibodies. The secondary antibody solution contained 1:1000 AF488-conjugated goat anti-chicken (Invitrogen A-11039) and 1:1000 AF647-conjugated goat anti-rabbit antibodies (Invitrogen A-32733) in blocking solution. From this point onwards, care was taken to prevent exposure of the tissue to light. After overnight incubation, on the final day, tissues were rinsed in PBS and counterstained with 1:5000 DAPI, before being mounted and sealed with nail polish. Stained slides were stored at 4 °C until imaged.

### Electrophysiology

Mice were deeply anesthetized with isoflurane, perfused transcardially with ice-cold cutting solution (in mM: 93 NMDG, 2.5 KCl, 1.2 NaH_2_PO_4_, 30 NaHCO_3_, 20 HEPES, 25 glucose, 5 sodium ascorbate, 2 thiourea, 3 sodium pyruvate, 10 MgSO_4_, 0.5 CaCl_2_, pH 7.3 adjusted with HCl, osmolarity 300–310 mOsm/kg) and subsequently decapitated. The brain was rapidly removed and coronal brain slices (400 µm thick; Leica VT1000S vibratome) were prepared in ice-cold cutting solution. Following dissection, slices were allowed to recover in oxygenated artificial cerebrospinal fluid (aCSF in mM: 118 NaCl, 25 NaHCO_3_, 10 glucose, 2.5 KCl 2.5, 1.2 NaHPO_4_, 1.3 MgCl_2_, 2.5 CaCl_2_) for 30 min at 32 °C, before equilibrating at room temperature for at least an additional 30 min. Slices were visualized using an upright microscope (Olympus BX50WI) and field potentials were recorded using a Multiclamp 700B amplifier (Molecular Devices). During recording, slices were perfused with heated aCSF (30 ± 2 °C). Recording pipettes were prepared from borosilicate glass (GC150F, Harvard Apparatus) and pulled to a tip resistance of 3–6 MΩ (Narishige PC-10) when filled with aCSF. Extracellular field potential in the subgranular layer of the dentate gyrus was evoked every 30 s, by the local electrical stimulation of the medial perforant pathway using a theta glass pipette. Correct placement in this pathway was confirmed by the observation of paired-pulse depression responses at an interpulse interval of 50 ms. An input/output (I/O) curve with stimulation intensities ranging from 0 to 8.0 V (in steps of 0.5 V), each applied three times with a 30–120 s interval, was then established. LTP was induced by a theta-burst protocol (10 trains at 5 Hz of 10 pulses at 100 Hz repeated 3 times, 20 s apart).

## Results

### Low-frequency scanning ultrasound penetrates the entire brain without adversely increasing tissue temperature

To first ensure accurate delivery of SUS, we placed the ketamine-anesthetized mouse in ear bars mounted on a heat pad. The 3D positioner-controlled ultrasound transducer TIPS system (Supplementary Fig. S2A) was then programmed to deliver the SUS treatment in a raster grid pattern that covered the entire cerebrum (Fig. 1A, B). To illustrate the accuracy of treatment delivery, an animal was intravenously injected with MBs, after which a restricted scanning pattern was delivered. Following the sonication, the mouse was intravenously injected with Evans blue to visualize the SUS path. Importantly, extravasation only occurred at the site of SUS as visualized by the neatly aligned Evans blue foci that followed the scanning pattern (Fig. 1C). By cutting a sonicated brain coronally into sections, we confirmed that the ultrasound had penetrated through the entire depth of the brain (Fig. 1D).

High-intensity focused ultrasound (HIFU) treatment is an FDA-approved procedure for ablating thalamic tissue to treat essential tremor [14]. This procedure results in an intended substantial temperature increase, which is not the case for the sonication protocol used in this study. The protocol described here and in our previous studies [3, 15] is conducted at a much lower frequency than that of HIFU. Nonetheless, in order to confirm the absence of an aversive temperature rise as a potential confound, we conducted thermal testing using a gray matter mimetic gel in both the presence and absence of a skull fragment (Supplementary Fig. S2B). We measured the temperature before and after SUS delivery for each protocol (Fig. 1E), and the TIPS system then provided the change in temperature during the experiment for each condition, revealing a maximal rise of ~1.1 °C at the skull (Fig. 1F). To confirm these findings, we also conducted a numerical estimate based on previous findings (Supplementary Text). While the phantom with skull experiment and numerical estimate matched well, with both revealing a ΔT of ~1.1 °C, for the gray matter tissue mimetic, ΔT was markedly higher at 0.22 °C for SUS in the presence of MBs (SUS^+MB^) compared to 0.046 °C for SUS in the absence of MBs (SUS^only^). The disparity between the numerical estimate and phantom measurements is likely due to the cooling effect of blood flow through living tissue, whereas the phantom has no such heat exchange mechanism. The slight temperature rise seen in the no-skull condition was also close to the detection limit of the thermocouple and may therefore be subject to tiny fluctuations in baseline temperature. Taken together, these estimations indicate that little tissue heating is experienced from the sonication procedure we adopted. Assuming a mouse body temperature of 36.8 °C [16], tissue heating would remain far below the tissue damage threshold of 43 °C [17], and therefore is not a factor in the findings presented here. As shown below, the absence of increases in heat-shock proteins in a quantitative proteomics analysis compared to sham further supports the notion that there are no heat-related bio-effects.

### Ultrasound treatment without microbubbles does not induce BBB opening in aged mice

**Fig. 2:**
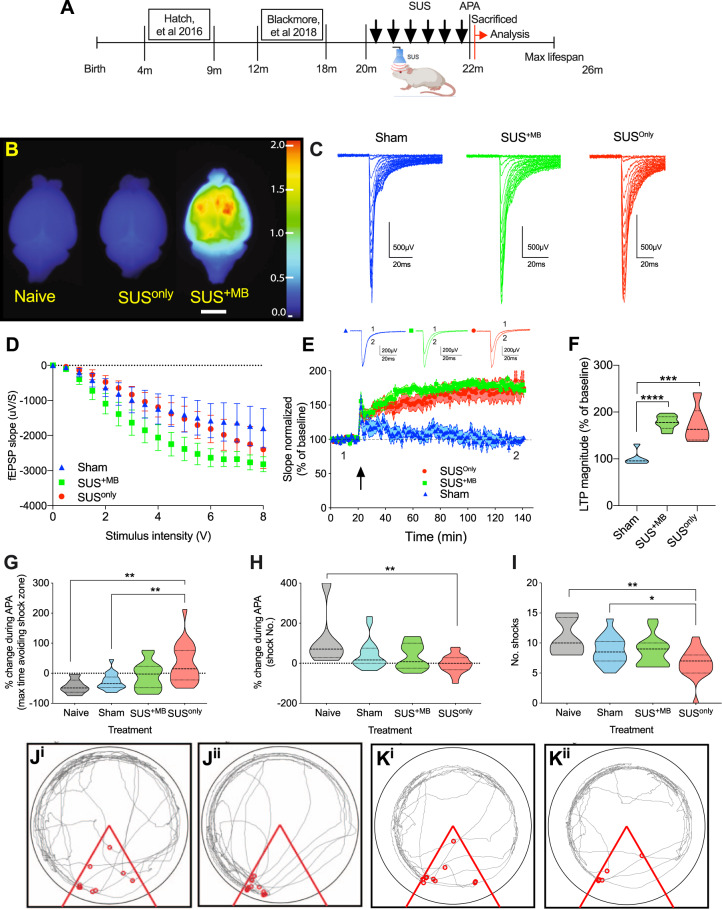
SUS treatment rescues LTP in the dentate gyrus of senescent animals and increases synaptic activity, and improves spatial learning ability. **A** Schematic highlighting the age of mice previously examined following SUS treatment, with this study examining the effect of SUS on aged, sedentary mice. After the final SUS treatment, animals underwent a single 30 min test on the active place avoidance (APA) test, comparing 22-month-old SUS^+MB^, SUS^only^, sham, and age-matched naive mice. **B** Naive mice or those that had undergone the SUS^only^ or SUS^+MB^ procedure were intravenously injected with Evans blue to visualize BBB opening. Whole mouse brains were scanned at 700 nm in the LiCor Odyssey scanner. The heat map shows relative fluorescence intensities, with higher intensities (see color bar) representing BBB opening. Scale bar: 5 mm. **C** Representative example of an input/output (I/O) curve for sham (mean = −944.9 ± 138.8 µV/s SEM), SUS^+MB^ (mean = −1762 ± 236.7 µV/s SEM), and SUS^only^ mice (mean = −1164 ± 197.1 µV/s SEM). **D** SUS treatment increased synaptic transmission only in the SUS^+MB^ group (one-way ANOVA [2.15 = 4.705, *p* = 0.0137, with Bonferroni post hoc analysis). **E** Following theta-burst stimulation (TBS) in vitro (indicated by a black arrow), no LTP was observed in the sham mice; however, LTP was fully rescued in both the SUS^+MB^ and SUS^only^ mice. **F** Histogram representing the average of the last 10 min of LTP for each treatment represented as a percentage of baseline, with significant increases observed in both the SUS^+MB^ and SUS^only^ groups (one-way ANOVA [*F*(2,17) = 19.85, *p* < 0.001], with Bonferroni post hoc analysis). *p < 0.05, **p < 0.01, ***p < 0.001, and ****p < 0.0001. **G** The SUS^only^ group exhibited an increase in the maximum time spent avoiding the aversive shock zone during testing relative to the naive, sham, and SUS^+MB^ treatment groups (one-way ANOVA [*F*(3,56) = 6.934), *p* = 0.0005], with Bonferroni post hoc analysis). **H** The SUS^only^ group had a significant reduction in the number of shocks in the last 5 min of testing relative to the first 5 min (one-way ANOVA [*F*(3,56) = 4.552), *p* = 0.0063], with Bonferroni post hoc analysis). **I** There was a significant reduction in the number of shocks the SUS^only^ group received compared to the sham group (one-way ANOVA [*F*(3,56) = 5.739), *p* = 0.001], with Bonferroni post hoc analysis). **J** Representative trace maps of a naive animal for the first 5 min (**J**^**i**^) and final 5 min (**J**^**ii**^) of the APA test, with red dots representing shocks received. **K** Representative trace maps of a SUS^only^ animal during the first 5 min (**K**^**i**^) and final 5 min (**K**^**ii**^) of the APA test. During the testing period there was a reduction in the number of shocks received following SUS^only^ treatment.

The SUS^+MB^ group (*n* = 15, aged 22 months at completion) received ultrasound treatments under ketamine anesthesia in the presence of intravenously injected MBs (Fig. 2A and Supplementary Fig. S1), using the TIPS system operated at a 1 MHz center frequency. An anesthetized sham group (*N* = 18, 22 months at completion) that received neither MBs nor SUS treatment was included as a control. To control for any potential confounds of ketamine, naive mice (*n* = 6, 22 months) were also included. We further performed ultrasound treatments in an age-matched anesthetized cohort which did not receive MBs (SUS^only^; *n* = 20, 22 months at completion), initially intended as a third control because our earlier studies had always used MBs to allow blood-borne factors to enter the brain and elicit bio-effects including amyloid and tau clearance [3, 18]. Although all groups (except for naive) underwent their respective ultrasound treatment six times, for simplicity, these groups will be referred to as SUS^+MB^, SUS^only^, and sham (Fig. 2A). Considering these differences and the advanced age of the mice, we also determined in a separate cohort of mice that BBB opening differed between the groups. Uptake of intravenously injected Evans blue, which binds to blood-borne and interstitial albumin (65 kDa), revealed that opening was only achieved following SUS^+MB^, and was not present under SUS^only^ or naive conditions, thereby ruling out increased, generalized BBB “leakage” with aging (Fig. 2B).

### Ultrasound treatment with and without BBB opening restores LTP induction in aged mice

We first asked whether ultrasound can restore the induction of LTP as an electrophysiological correlate of memory in aged (22-month-old) mice [19]. We obtained hippocampal slices and tested LTP in the medial perforant path input 24 h after APA testing had been conducted. Perforant path input to the dentate gyrus did not differ between the two SUS treatments as compared to sham animals, as shown by the I/O curves (Fig. 2C, D). After a 20 min baseline, LTP was induced using theta-burst stimulation. In slices prepared from young animals, theta-burst stimulation evokes robust LTP [2]. As reported previously for old mice [20], no LTP was evident in the sham group. However, to our surprise, LTP induction was rescued in both the SUS^+MB^ and SUS^only^ groups (Fig. 2E, F). This demonstrates that in the physiologically aged brain, BBB opening, i.e., blood-borne factors entering the brain, is not required for the observed restorative effects of SUS.

### SUS^only^ treatment improves spatial learning ability in aged mice

We next determined whether repeated sessions of therapeutic ultrasound restore spatial memory in aged WT mice, analyzing four groups of animals. The treatment was followed by behavioral testing using an accelerated APA paradigm, investigating the animals’ performance in 5 min intervals. The maximum time spent avoiding the shock zone increased in the SUS^only^ group, but not in the SUS^+MB^ cohort compared to both the naive and sham groups (Fig. 2G). Similarly, the SUS^only^ group showed improved learning ability as calculated by a reduction in the number of shocks in the last 5 min relative to the first 5 min of testing (Fig. 2H), and a reduction in the number of shocks at the end of the testing period (Fig. 2I). Sham and naive mice behaved similarly, suggesting that anesthesia had no effect on memory. Representative trace maps for the first 5 min of the test in naive (Fig. 2J^i^) and SUS^only^ mice (Fig. 2K^i^) compared to the last 5 min (Fig. 2J^ii^ and K^ii^, respectively) revealed that the SUS^only^ animals learned more effectively to avoid the aversive shock zone. The SUS^+MB^ paradigm showed a trend toward improved cognition, whilst SUS^only^ resulted in statistically significant improvements in spatial learning.

### Evidence for improved function of synaptic NMDA receptors induced by ultrasound

**Fig. 3:**
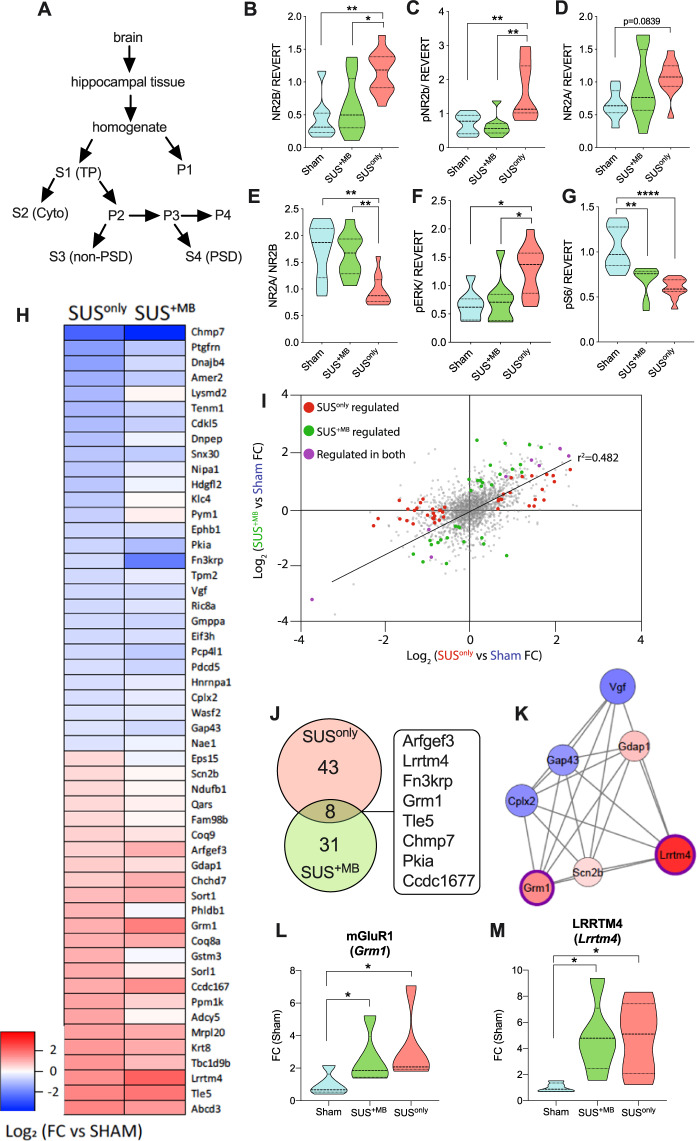
SUS treatment alters the levels and activities of hippocampal proteins in the postsynaptic density fraction and alters the proteomic profile of hippocampal proteins in the total protein fraction. **A** Schematic illustrating how specific fractions were obtained from hippocampal samples to conduct biochemical analyses. **B** NR2B and **C** pNR2B were increased in postsynaptic fractions from SUS^only^ mice compared to the sham and SUS^+MB^ groups. **D** NR2A levels were not altered following SUS treatment. **E** The NR2A/2B ratio was reduced in SUS^only^-treated animals. **F** There was a significant increase in pERK for SUS^only^ animals. **G** Both SUS^+MB^ and SUS^only^ treatments produced decreases in pS6 levels relative to sham-treated animals (one-way ANOVA with Bonferroni post hoc analysis). **H** Heat map of proteins significantly altered following SUS^only^ treatment (|FC| ≥ 1.5, *p* ≤ 0.05). **I** Comparison of SUS^+MB^ and SUS^only^ treatment groups reveals that the vast majority of regulated proteins (72/82) show similar directional changes in fold-change relative to sham controls (Pearson’s correlation, *r*^2^ = 0.482). **J** Venn diagram of proteins significantly altered in abundance in SUS^+MB^ and SUS^only^ treatment groups compared to sham controls (|FC| ≥ 1.5, *p* ≤ 0.05). **K** STRING analysis revealed a cluster associated with synaptic signaling, including increases in both *mGluR1* (**L**) and *Lrrtm4* (**M**), after both SUS^+MB^ and SUS^only^ treatment (one-way ANOVA with Bonferroni post hoc analysis). *p < 0.05, **p < 0.01, and ****p < 0.0001.

At the synaptic level, age-dependent deficits in hippocampal learning and LTP have been associated with changes in synaptic receptor composition, which impair effective neuronal communication [21]. We therefore next examined whether the two SUS treatment paradigms altered the levels and activities of key proteins in the hippocampus compared to sham. The PSD and TP fractions were obtained from dissected hippocampi (Fig. 3A) and analyzed by western blotting (Supplementary Figs. S3 and S4). In the purified PSD fraction, we found that SUS^only^ treatment increased the levels of NR2B (Fig. 3B), a component of mature NMDA receptors that is required for LTP induction at glutamatergic synapses [22, 23]. Phosphorylation of NR2B (pNR2B) by the kinase CaMKII has been found to play a critical role in learning and memory storage [24], and importantly, pNR2B levels were also increased following the SUS^only^ treatment (Fig. 3C). No change was observed for NR2A (Fig. 3D), but the NR2A/B ratio was significantly reduced after SUS^only^ treatment (Fig. 3E). Lowering this ratio has been shown to reduce the threshold for LTP induction in the murine visual cortex [25]. The kinase ERK, which is required for modulating synaptic plasticity [26], showed a trend toward increased levels (Supplementary Fig. S5A), whereas levels of phosphorylated ERK were significantly increased in the SUS^only^ group (Fig. 3F). Levels of the ribosomal protein S6 (Supplementary Fig. S5B) and pS6 (Fig. 3G) were decreased following SUS treatment. However, this was different from the total fraction in which increases in S6 and pS6 levels were observed (Supplementary Fig. S6G, H). This is not surprising considering that in active synapses, S6 is localized to the activated dendritic membrane contacted by such synapses to induce protein synthesis [27]. Additional changes in the hippocampal PSD and total fractions are shown in Supplementary Figs. S5C and S6A–F. Taken together these findings demonstrate that therapeutic ultrasound leads to significant, activating changes in synaptic signaling.

### Quantitative proteomics further highlights increased synaptic plasticity following ultrasound treatment

We next aimed to investigate these changes in an unbiased manner. Focusing on the effects of SUS^only^ and SUS^+MB^ treatment compared to sham, we therefore performed a quantitative proteomic analysis using SWATH-MS on total hippocampal fractions (Fig. 3H–M). Under SUS^only^ conditions, the abundance of 51 proteins was significantly altered compared to sham (Fig. 3H, FC| ≥ 1.5, *p* ≤ 0.05), whereas for SUS^+MB^ we identified 39 regulated proteins, of which 21 were increased and 18 decreased (Fig. 3I and Supplementary Table S2 for complete list). Interestingly, for the vast majority (88%) of the regulated proteins, the fold-change compared to sham was similar across both treatment groups (*r*^2^ = 0.482) (Fig. 3I), with SUS^+MB^ producing a greater degree of variation (Supplementary Fig. S7A). Many of the proteins found to be altered in response to SUS^only^ were associated with synaptic function and neuronal growth/neurogenesis, including glutamate metabotropic receptor 1 (*Grm1*:mGluR1), cyclin-dependent kinase-like 5 (*CDKL5*), ephrin type-B receptor 1 (*EPHB1*), neurosecretory protein VGF (*Vgf*), complexin-2 (*Cplx2*), and neuromodulin (*Gap43*) (Supplementary Table S2), further supporting the conclusion that SUS treatment leads to synaptic changes. GO analysis also suggested that proteins associated with intracellular sorting and mitochondrial function were affected by the SUS treatment (Supplementary Fig. S7B), both of which are required for successful neuronal function.

Eight proteins that exhibited either an increase or a decrease compared to sham were shared between the SUS^only^ and SUS^+MB^ treatment groups (Fig. 3J), with increases found for brefeldin A-inhibited guanine nucleotide-exchange protein 3 (*Arfgef3*), leucine-rich repeat transmembrane neuronal protein 4 (*Lrrtm4*), mGluR1, TLE family member 5 (*Tle-*5), and coiled-coil domain-containing protein 167 (*Ccdc167*), and decreases in ketosamine-3-kinase (*Fn3krp*), charged multivesicular body protein 7 (*Chmp7*), and cAMP-dependent protein kinase inhibitor alpha (*Pkia*). Changes in these proteins are associated with the regulation of excitatory synapse development, the induction of LTP, and long-term depression (LTD), embryonic neurogenesis, endosomal sorting, protein deglycation, and cAMP-dependent protein kinase signaling (Supplementary Table S2). These shared protein changes likely explain our finding that SUS treatment, either with or without the addition of MBs, restores LTP in the aged murine brain.

STRING network analysis of proteins that were altered in the SUS^only^ treatment group identified a cluster of proteins associated with synaptic signaling (Fig. 3K), including an increase in both mGluR1 (GRM1) (Fig. 3L) that has been shown to be associated with spatial learning [28] and *Lrrtm4*, which is critically involved in synapse development [29] (Fig. 3M). These data again support the notion that SUS^only^ treatment enhances synaptic plasticity, ultimately culminating in improved spatial learning ability.

The proteomics dataset was also used, as an additional assessment of any potential increase in heat during the sonication procedure, to determine whether levels of heat-shock proteins were altered. Assessment of 24 heat-shock proteins in SUS^+MB^ and SUS^only^ conditions compared to sham revealed no significant differences in protein levels (Supplementary Fig. S7C), further supporting the notion that increased heat is not a contributing factor to the results reported here.

### SUS^only^ induces alterations to the extracellular matrix

**Fig. 4:**
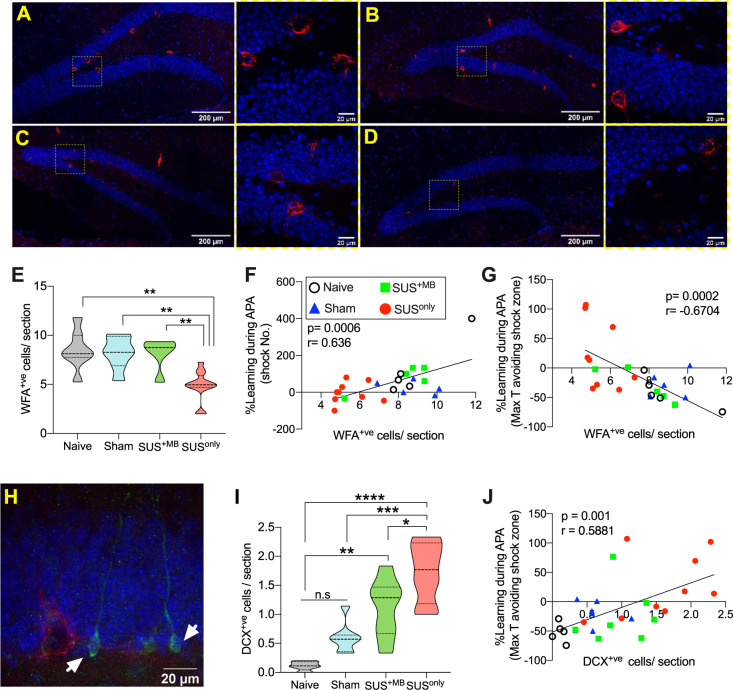
SUS treatment reduces the extracellular matrix and increases neurogenesis in aged animals. **A**–**D** WFA staining (in red, DAPI in blue) of brain sections from naive (**A**), sham (**B**), SUS^+MB^ (**C**), and SUS^only^ mice (**D**). **E** There was a significant decrease in WFA^+ve^ cells in the dentate gyrus of animals treated with SUS^only^ (one-way ANOVA [*F*(3,27) = 9.963, *p* = 0.0001], with Bonferroni post hoc analysis). **F** A significant positive correlation was observed between the number of WFA^+ve^ cells and learning ability in the APA task and the change in shock numbers (**G**). **H** Representative photomicrograph showing DCX^+ve^ cells (in green, WFA in red) in the dentate gyrus of naive animals. **I** SUS^+MB^ mice exhibited an increase in the number of DCX^+ve^ cells/section compared to naive animals, whereas SUS^only^ animals had significantly more DCX^+ve^ cells/section than all other treatment groups (one-way ANOVA [*F*(3,23) = 18.37, *p* < 0.0001], with Bonferroni post hoc analysis). **J** There was a significant positive correlation between the number of DCX^+ve^ cells/section and learning ability in the APA task. *p < 0.05, **p < 0.01, ***p < 0.001, and ****p < 0.0001.

Synaptic plasticity and cognitive function are both associated with alterations in PNNs, a major component of the extracellular matrix of the brain [30]. When PNNs are transiently removed in either aged WT or AD mice, cognitive ability improves [31]. Because ultrasound waves generated by SUS constitute a pressure wave, we reasoned that this may be a signal to disrupt or break down the extracellular matrix, thereby contributing to enhanced cognition. We therefore examined changes in PNN distribution via immunolabeling using the lectin WFA. This revealed a significant reduction in WFA-positive (WFA^+ve^) cells in the dentate gyrus in SUS^only^ mice compared to naive, sham or SUS^+MB^ animals (Fig. 4A–E). In support of a potential contribution of PNNs to the cognitive restoration observed following SUS treatment, significant correlations could be established between the number of WFA^+ve^ cells and APA performance (Fig. 4F, G).

### SUS treatment increases neurogenesis in aged mice

Changes to the extracellular matrix are also tightly linked to neurogenesis and neuronal plasticity [32]. Not surprisingly, adult hippocampal neurogenesis is required for spatial learning [33], with the ablation of immature neurons severely compromising hippocampal-dependent cognitive ability [34]. When neurogenesis, which decreases in mice during aging [35] and is virtually nonexistent by 22 months, is experimentally induced either by environmental enrichment or exercise, spatial memory functions are restored [9]. Similarly, the induction of hippocampal LTP is transiently enhanced in maturing new-born adult neurons [22]. Considering that the SUS^+MB^ and SUS^only^ groups both displayed restored LTP induction and, to different degrees, hippocampal spatial learning, we assessed neurogenesis by examining the number of new-born DCX^+ve^ cells in the dentate gyrus [36]. This revealed that SUS^+MB^ increased the number of DCX^+ve^ cells 7-fold compared to age-matched naive animals, an increase similar to that which has been reported previously by achieving focal BBB opening with ultrasound [37–39]. The largest increase in neurogenesis, however, was observed in the SUS^only^ group, which exhibited a 13-fold increase in DCX^+ve^ cells compared to the naive group (Fig. 4H, I). Significant correlations could also be established between the number of DCX^+ve^ cells and performance in the APA test (Fig. 4J).

### SUS treatment and astrocytes

Our study so far emphasizes improved function of synaptic NMDA receptors induced by ultrasound. A recent study investigated the role of astrocytes in neuromodulation [40]. The study reported a role for TRPA1 channels in astrocytes resulting in the entry of Ca^2+^ ions in an acute response to ultrasound, with the consequence of glutamate release activating NMDARs. For this reason, we assessed the hippocampal fractions from sham, SUS^+MB^ and SUS^only^ treatments for TRPA1, Gap43, and serine racemase (Supplementary Fig. S8). Whereas the two latter proteins did not reveal significant differences, for TRPA1 we found a significant increase for the SUS^only^ condition compared to sham and a trend compared to SUS^+MB^ two days after treatment (Supplementary Fig. S8A). We also analyzed GFAP by immunofluorescence and found a significant increase in % fluorescence in SUS^+MB^-treated animals compared to the other treatments. However, we saw no difference in Iba1-positive cells or in microglial branching pattern between treatments (Supplementary Fig. S8F, G).

### Timecourse of memory improvement after SUS^only^ treatment suggests progressive improvements with treatment sessions

**Fig. 5:**
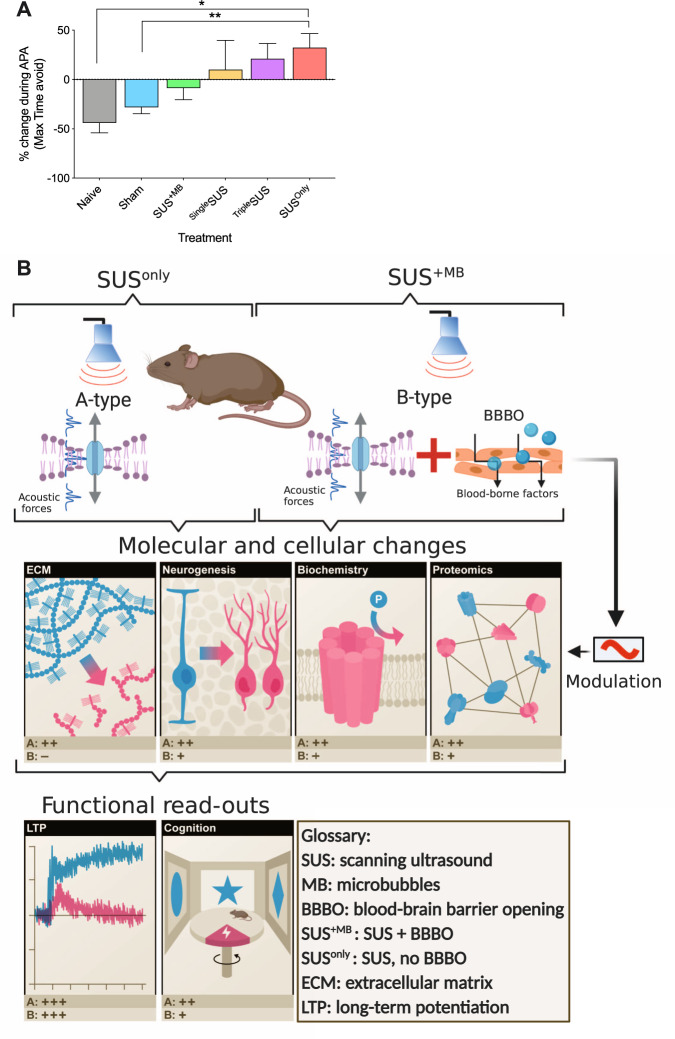
Timecourse of memory improvements after SUS^only^ treatment and integrated model of the effects of SUS on the aging brain. **A** Comparing the change in the maximum time animals can avoid the shock zone as a measure of the learning ability during APA reveals a trend toward increased ability after one (^Single^SUS, *n* = 8) and three SUS^only^ treatments (^Triple^SUS, *n* = 8), which after six SUS^only^ sessions (SUS^only^, *n* = 20) becomes significant (one-way ANOVA [*F*(5,70) = 3.879, *p* = 0.0037] with Bonferroni post hoc analysis). **B** Integrated model differentiating A-type effects of SUS due to a pressure wave (SUS^only^) and B-type effects, adding modulatory effects due to BBB opening and the uptake of blood-borne factors by the brain (SUS^+MB^). The acoustic forces from SUS alter the hippocampal milieu by reducing the ECM, increasing neurogenesis and altering the activity and proteomic profile of hippocampal proteins. These changes ultimately result in improved functional outcomes including reinstatement to induce LTP and improved spatial learning. Scoring (pluses and minuses) indicates the corresponding effect for the respective read-outs.

Our analysis suggests that SUS improves memory functions and LTP induction via a pleiotropic mode of action. Another important question is whether the changes in memory functions, underpinned by cellular and biochemical changes, occur gradually or immediately after acute treatment. We therefore determined the effect of sonication without MBs after one session (^Single^SUS, *N* = 8) or three sessions (^Triple^SUS, *N* = 8 and see Supplementary Fig. S1) compared to the six treatment sessions used throughout this study (SUS^only^, *N* = 20). This revealed a trend toward an increased performance with increased session numbers (Fig. 5A); however, we only observed a significant spatial memory improvement after six sessions (see Supplementary Table S1). These results suggest that, in the aged brain, improvements in spatial learning occur over multiple SUS treatment sessions.

## Discussion

One of the cardinal features of physiological aging is the deterioration of information processing, which presents, for example, as progressive impairments in learning and memory. In the C57Bl/6 mouse strain, senescence is reached at around 18 months of age [41], with a maximal lifespan of 24–26 months. In our study, we explored therapeutic ultrasound in these mice, initiating treatments at 20 months of age and continuing over a period of up to 6 weeks (Fig. 2A). Our data collectively suggest that therapeutic ultrasound can mitigate several aspects of age-associated cognitive decline. Whereas 18-month-old mice exhibit LTP and spatial memory formation [2], beyond this age, they become progressively impaired until they fail to form LTP. We have shown here that ultrasound with and without MBs is restorative in senescent mice, with both separate and overlapping effects. In both the SUS^only^ and SUS^+MB^ conditions (with the latter having an added effect of BBB opening due to cavitation), ultrasound acts as a pressure wave (radiation force) that exerts mechanical and mechano-electric effects that affect internal and cytoplasmic membranes of various cell-types and protein complexes, including channel proteins [42]. The radiation force is the unifying feature of our SUS^+MB^ and SUS^only^ sonication paradigms (Fig. 5B).

While our study, which predominantly focuses on neurons and the hippocampus as a brain area critically involved in memory and learning, suggests pleiotropic effects, it also highlights a particular role for an improvement in the function of synaptic NMDA receptors in response to ultrasound. The question that then arises is what mediates these effects on NMDA receptors. Interestingly, a recent study by Oh et al. [40] investigating the role of astrocytes (which are pressure sensitive) in response to ultrasound may provide an answer. Using a sonication paradigm similar to our SUS^only^ paradigm, their study investigated the neuromodulatory effects of low-intensity focused ultrasound. As an underlying mechanism, they reported a TRPA1-Best1-NMDA receptor pathway that complements our findings. The authors showed that ultrasound caused an opening of TRPA1 channels in astrocytes, resulting in the entry of Ca^2+^ ions. This in turn led to the release of gliotransmitters including glutamate through Best1 channels. The released glutamate was found to activate NMDA receptors in neighboring neurons, thereby eliciting action potential firing. Interestingly, we found in our study that the SUS^only^ paradigm significantly increased TRPA1 levels in synaptosomal hippocampal fractions compared to those from SUS^+MB^ or sham treatments. This increase, reflected by the changes observed for NMDA receptor subunits in these fractions, suggests that ultrasound-mediated astrocytic glutamate release is a likely mechanism by which SUS leads to the observed improvements in senescent mice.

These findings do not, however, rule out additional mechanisms that could, for example, involve neurogenesis [43, 44]. Of note, we found that SUS^+MB^ increased the number of DCX^+ve^ cells in the dentate gyrus 7-fold whereas SUS^only^ led to a 13-fold increase in comparison with age-matched naive animals. Similarly, the extracellular matrix has been proposed to have a role in neuronal functions including memory, with the digestion, blocking or removal of PNNs influencing functional recovery after a variety of central nervous system lesions [30, 45]. Depletion of perineuronal nets has been shown to enhance recognition memory and LTD in the perirhinal cortex in mice [46]. Similarly, our analysis revealed a significant reduction in WFA^+ve^ cells in the dentate gyrus in SUS^only^ mice compared to either naive, sham, or SUS^+MB^ animals. The absence of a reduction in the SUS^+MB^ condition does not exclude the reduction of other components of the extracellular matrix. Furthermore, whilst we cannot rule out the possibility that inflammatory factors contribute to the observed effects, we did not observe changes to microglial morphology between treatments.

What are the fundamental differences between the SUS^only^ and SUS^+MB^ outcomes in physiologically aged mice? It is important to note that both procedures had no detrimental effect but rather remained unaltered or resulted in improvements in different parameters relative to both naive and sham controls. Both paradigms, SUS^only^ (radiation force) and SUS^+MB^ (radiation plus cavitation force) produced beneficial effects, albeit to different degrees (Fig. 5B). They resulted in a similar induction of LTP that is not possible in mice of this age that undergo sham treatments. Spatial memory, on the other hand, was restored at statistically significant levels only in the SUS^only^ condition. Neurogenesis was very strongly induced in both conditions; however, the extracellular matrix was only reduced under SUS^only^ conditions although a detailed analysis going beyond WFA counts was not performed. The fractionation of hippocampal tissue revealed pronounced effects for SUS^only^, with SUS^+MB^ mostly showing a trend in the same direction. Levels of pS6 were significantly reduced in both conditions compared to sham. Our proteomic analysis, which by its nature is unbiased, revealed uniform changes in synaptic proteins for both sonication paradigms. Two proteins (mGluR1 and LRRTM4) were increased under both SUS^only^ and SUS^+MB^ conditions. Therefore, the difference in therapeutic outcomes between the two experimental paradigms may reflect issues of sensitivity. It is also conceivable that with an opened BBB under SUS^+MB^ conditions not only can blood-borne factors enter the brain but these factors crucial for the observed effects may also be cleared from the brain. Moreover, because astrocytosis was increased after the SUS^+MB^ treatment, whereas the SUS^only^ procedure resulted in increased TRPA1 levels, this may suggest that astrocytes respond differentially to the two treatment paradigms. Further elucidation of these and other differences between the SUS^+MB^ and SUS^only^ paradigms will be the subject of future studies.

What are the implications of our studies in the context of earlier work done in AD mouse models? The SUS^+MB^ approach referred to here is the technique we have previously employed to demonstrate efficacy in clearing protein aggregates from the transgenic AD mouse brain also using the TIPS system [3, 4]. WT mice do not naturally develop aggregates and tangles, which are hallmarks of AD pathology. They do, however, develop cognitive deficits during the course of aging. Our results revealed that although SUS^+MB^ improved certain parameters within the hippocampus, SUS^only^ was optimal in this experimental setting. This suggests that SUS^+MB^ within AD mouse models acts first to activate microglia that in turn clears protein aggregates. This then allows functionality to be returned via multiple mechanisms, likely by creating a more permissive environment for the formation of new synapses and allowing more efficient communication between neurons (and astrocytes) within the brains of these animals. In non-pathological animals, protein aggregates do not need to be removed, and SUS^only^ is able to create an environment that includes the activation of astrocytes and neurons, increased neurogenesis, and improved synaptic efficiency, all of which contribute to the observed restoration of cognitive function.

What are the implications of our study for human conditions of memory impairment? Whereas therapeutic ultrasound with MBs (SUS^+MB^) is currently being explored as a treatment modality for AD because of its effects on clearing protein aggregates due to the uptake of blood-borne therapeutic factors [3, 4, 47], our data suggest an additional benefit by restoring LTP with ultrasound only (SUS^only^). Our work adds to several studies that highlight the neuromodulatory potential of therapeutic ultrasound in different species and experimental paradigms [48–52]. With the observed changes associated with synaptic plasticity, SUS may therefore be an additional non-invasive modality, as to some extent shown for repeated transmagnetic stimulation, transdural electric nerve stimulation, and direct current stimulation, to induce LTP or LTP-like plasticity, which have been suggested to hold promise for the treatment of a variety of neurological conditions, including neuropathic pain, epilepsy, depression, tinnitus, and stroke [53]. Here we performed a comparative analysis of senescent mice subjected to a treatment scheme that either uses MBs to achieve BBB opening or operates without BBB opening, followed by an analysis of the underlying cellular and molecular changes. In a clinical setting, the SUS^only^ treatment modality may provide (i) potential benefits in improving treatment safety (because the oscillation of MBs induced by ultrasound can lead to inertial cavitation associated with tissue damage) and (ii) the possibility of using ultrasound on its own to enhance cognition in physiologically aging humans.

## Supplementary information

Supplementary Figures S1–S8/Supplementary Text

Supplementary Table S1

Supplementary Table S2

## References

[1] Lopez-Otin C, Blasco MA, Partridge L, Serrano M, Kroemer G (2013). The hallmarks of aging. Cell..

[2] Blackmore DG, Turpin F, Mohamed AZ, Zong F, Pandit R, Pelekanos M (2018). Multimodal analysis of aged wild-type mice exposed to repeated scanning ultrasound treatments demonstrates long-term safety. Theranostics..

[3] Leinenga G, Götz J (2015). Scanning ultrasound removes amyloid-beta and restores memory in an Alzheimer’s disease mouse model. Sci Tran Med.

[4] Nisbet RM, Van der Jeugd A, Leinenga G, Evans HT, Janowicz PW, Götz J (2017). Combined effects of scanning ultrasound and a tau-specific single chain antibody in a tau transgenic mouse model. Brain..

[5] Leinenga G, Langton C, Nisbet R, Götz J (2016). Ultrasound treatment of neurological diseases—current and emerging applications. Nat Rev Neurol.

[6] Pandit R, Koh WK, Sullivan RKP, Palliyaguru T, Parton RG, Götz J (2020). Role for caveolin-mediated transcytosis in facilitating transport of large cargoes into the brain via ultrasound. J Control Release.

[7] Turturro A, Duffy P, Hass B, Kodell R, Hart R (2002). Survival characteristics and age-adjusted disease incidences in C57BL/6 mice fed a commonly used cereal-based diet modulated by dietary restriction. J Gerontol A Biol Sci Med Sci.

[8] Benice TS, Rizk A, Kohama S, Pfankuch T, Raber J (2006). Sex-differences in age-related cognitive decline in C57BL/6J mice associated with increased brain microtubule-associated protein 2 and synaptophysin immunoreactivity. Neuroscience..

[9] van Praag H, Shubert T, Zhao C, Gage FH (2005). Exercise enhances learning and hippocampal neurogenesis in aged mice. J Neurosci.

[10] Seip R, Chin CT, Hall CS, Raju BI, Ghanem A, Tiemann K (2010). Targeted ultrasound-mediated delivery of nanoparticles: on the development of a new HIFU-based therapy and imaging device. IEEE Trans Biomed Eng.

[11] Li C, Götz J (2017). Somatodendritic accumulation of Tau in Alzheimer’s disease is promoted by Fyn-mediated local protein translation. EMBO J.

[12] Evans HT, Benetatos J, van Roijen M, Bodea LG, Götz J. Decreased synthesis of ribosomal proteins in tauopathy revealed by non-canonical amino acid labelling. EMBO J. 2019;38:e101174. 10.15252/embj.2018101174 PMC6600635 31268600

[13] Ge SX, Jung D, Yao R (2020). ShinyGO: a graphical gene-set enrichment tool for animals and plants. Bioinformatics..

[14] Fishman PS, Elias WJ, Ghanouni P, Gwinn R, Lipsman N, Schwartz M (2018). Neurological adverse event profile of magnetic resonance imaging-guided focused ultrasound thalamotomy for essential tremor. Mov Disord.

[15] Pandit R, Leinenga G, Götz J (2019). Repeated ultrasound treatment of tau transgenic mice clears neuronal tau by autophagy and improves behavioral functions. Theranostics..

[16] Refinetti R (2010). The circadian rhythm of body temperature. Front Biosci (Landmark Ed).

[17] Yarmolenko PS, Moon EJ, Landon C, Manzoor A, Hochman DW, Viglianti BL (2011). Thresholds for thermal damage to normal tissues: an update. Int J Hyperth.

[18] Hatch RJ, Leinenga G, Götz J (2016). Scanning ultrasound (SUS) causes no changes to neuronal excitability and prevents age-related reductions in hippocampal CA1 dendritic structure in wild-type mice. PLoS One.

[19] Malenka RC (2003). The long-term potential of LTP. Nat Rev Neurosci.

[20] Froc DJ, Eadie B, Li AM, Wodtke K, Tse M, Christie BR (2003). Reduced synaptic plasticity in the lateral perforant path input to the dentate gyrus of aged C57BL/6 mice. J Neurophysiol.

[21] Nicoll RA (2017). A brief history of long-term potentiation. Neuron..

[22] Ge S, Yang CH, Hsu KS, Ming GL, Song H (2007). A critical period for enhanced synaptic plasticity in newly generated neurons of the adult brain. Neuron..

[23] Foster KA, McLaughlin N, Edbauer D, Phillips M, Bolton A, Constantine-Paton M (2010). Distinct roles of NR2A and NR2B cytoplasmic tails in long-term potentiation. J Neurosci.

[24] Bayer KU, Schulman H (2019). CaM kinase: still inspiring at 40. Neuron..

[25] Philpot BD, Cho KK, Bear MF (2007). Obligatory role of NR2A for metaplasticity in visual cortex. Neuron..

[26] Cohen-Matsliah SI, Seroussi Y, Rosenblum K, Barkai E (2008). Persistent ERK activation maintains learning-induced long-lasting modulation of synaptic connectivity. Learn Mem.

[27] Pirbhoy PS, Farris S, Steward O (2016). Synaptic activation of ribosomal protein S6 phosphorylation occurs locally in activated dendritic domains. Learn Mem.

[28] Conquet F, Bashir ZI, Davies CH, Daniel H, Ferraguti F, Bordi F (1994). Motor deficit and impairment of synaptic plasticity in mice lacking mGluR1. Nature..

[29] Siddiqui TJ, Tari PK, Connor SA, Zhang P, Dobie FA, She K (2013). An LRRTM4-HSPG complex mediates excitatory synapse development on dentate gyrus granule cells. Neuron..

[30] Fawcett JW, Oohashi T, Pizzorusso T (2019). The roles of perineuronal nets and the perinodal extracellular matrix in neuronal function. Nat Rev Neurosci.

[31] Yang S, Cacquevel M, Saksida LM, Bussey TJ, Schneider BL, Aebischer P (2015). Perineuronal net digestion with chondroitinase restores memory in mice with tau pathology. Exp Neurol.

[32] Su W, Matsumoto S, Sorg B, Sherman LS (2019). Distinct roles for hyaluronan in neural stem cell niches and perineuronal nets. Matrix Biol.

[33] Vukovic J, Borlikova GG, Ruitenberg MJ, Robinson GJ, Sullivan RK, Walker TL (2013). Immature doublecortin-positive hippocampal neurons are important for learning but not for remembering. J Neurosci.

[34] Clelland CD, Choi M, Romberg C, Clemenson GD, Fragniere A, Tyers P (2009). A functional role for adult hippocampal neurogenesis in spatial pattern separation. Science..

[35] Kuhn HG, Dickinson-Anson H, Gage FH (1996). Neurogenesis in the dentate gyrus of the adult rat: age-related decrease of neuronal progenitor proliferation. J Neurosci.

[36] Couillard-Despres S, Winner B, Schaubeck S, Aigner R, Vroemen M, Weidner N (2005). Doublecortin expression levels in adult brain reflect neurogenesis. Eur J Neurosci.

[37] Mooney SJ, Shah K, Yeung S, Burgess A, Aubert I, Hynynen K (2016). Focused ultrasound-induced neurogenesis requires an increase in blood-brain barrier permeability. PLoS One.

[38] Shin J, Kong C, Lee J, Choi BY, Sim J, Koh CS (2019). Focused ultrasound-induced blood-brain barrier opening improves adult hippocampal neurogenesis and cognitive function in a cholinergic degeneration dementia rat model. Alzheimer’s Res Ther.

[39] Scarcelli T, Jordao JF, O’Reilly MA, Ellens N, Hynynen K, Aubert I (2014). Stimulation of hippocampal neurogenesis by transcranial focused ultrasound and microbubbles in adult mice. Brain Stimul.

[40] Oh SJ, Lee JM, Kim HB, Lee J, Han S, Bae JY (2019). Ultrasonic neuromodulation via astrocytic TRPA1. Curr Biol.

[41] Dutta S, Sengupta P (2016). Men and mice: relating their ages. Life Sci.

[42] Tyler WJ, Lani SW, Hwang GM (2018). Ultrasonic modulation of neural circuit activity. Curr Opin Neurobiol.

[43] Alam MJ, Kitamura T, Saitoh Y, Ohkawa N, Kondo T, Inokuchi K (2018). Adult neurogenesis conserves hippocampal memory capacity. J Neurosci.

[44] Deng W, Aimone JB, Gage FH (2010). New neurons and new memories: how does adult hippocampal neurogenesis affect learning and memory?. Nat Rev Neurosci.

[45] Senkov O, Andjus P, Radenovic L, Soriano E, Dityatev A (2014). Neural ECM molecules in synaptic plasticity, learning, and memory. Prog Brain Res.

[46] Romberg C, Yang S, Melani R, Andrews MR, Horner AE, Spillantini MG (2013). Depletion of perineuronal nets enhances recognition memory and long-term depression in the perirhinal cortex. J Neurosci.

[47] Lipsman N, Meng Y, Bethune AJ, Huang Y, Lam B, Masellis M (2018). Blood-brain barrier opening in Alzheimer’s disease using MR-guided focused ultrasound. Nat Commun.

[48] Pouget P, Frey S, Ahnine H, Attali D, Claron J, Constans C (2020). Neuronavigated repetitive transcranial ultrasound stimulation induces long-lasting and reversible effects on oculomotor performance in non-human primates. Front Physiol.

[49] Sanguinetti JL, Hameroff S, Smith EE, Sato T, Daft CMW, Tyler WJ (2020). Transcranial focused ultrasound to the right prefrontal cortex improves mood and alters functional connectivity in humans. Front Hum Neurosci.

[50] Brinker ST, Preiswerk F, White PJ, Mariano TY, McDannold NJ, Bubrick EJ (2020). Focused ultrasound platform for investigating therapeutic neuromodulation across the human hippocampus. Ultrasound Med Biol.

[51] Downs ME, Lee SA, Yang G, Kim S, Wang Q, Konofagou EE (2018). Non-invasive peripheral nerve stimulation via focused ultrasound in vivo. Phys Med Biol.

[52] Davidson B, Hamani C, Rabin JS, Goubran M, Meng Y, Huang Y (2020). Magnetic resonance-guided focused ultrasound capsulotomy for refractory obsessive compulsive disorder and major depressive disorder: clinical and imaging results from two phase I trials. Mol Psychiatry.

[53] Bliss TV, Cooke SF (2011). Long-term potentiation and long-term depression: a clinical perspective. Clinics (Sao Paulo).

