# Supplementary material for: Low-intensity ultrasound restores long-term potentiation and memory in senescent mice through pleiotropic mechanisms including NMDAR signaling
Source: Mol Psychiatry. 2021 May 27;26(11):6975–91. doi: 10.1038/s41380-021-01129-7 (PMC8760044; doi:10.1038/s41380-021-01129-7)
Supplement: Supplementary file 1 — Supplementary Figures S1–S8/Supplementary Text [file 41380_2021_1129_MOESM1_ESM.pdf]

Supplementary Information for

Low-intensity ultrasound restores long-term potentiation and memory in senescent mice through pleiotropic mechanisms including NMDAR signaling

Daniel G. Blackmore[1,†], Fabrice Turpin[2,†], Tishila Palliyaguru[1], Harrison T. Evans[1], Antony Chicoteau[1], Wendy Lee[1], Matthew Pelekanos[1], Nghia Nguyen[1], Jae Song[1], Robert K. P. Sullivan[2], Pankaj Sah[2,3], Perry F. Bartlett[1,2,3], Jürgen Götz[1,*]

Jürgen Götz; e-mail: j.goetz@uq.edu.au

This PDF file includes:

Supplementary Figures S1 to S8
Supplementary Tables S1 to S2
Supplementary text

Supplementary Figures

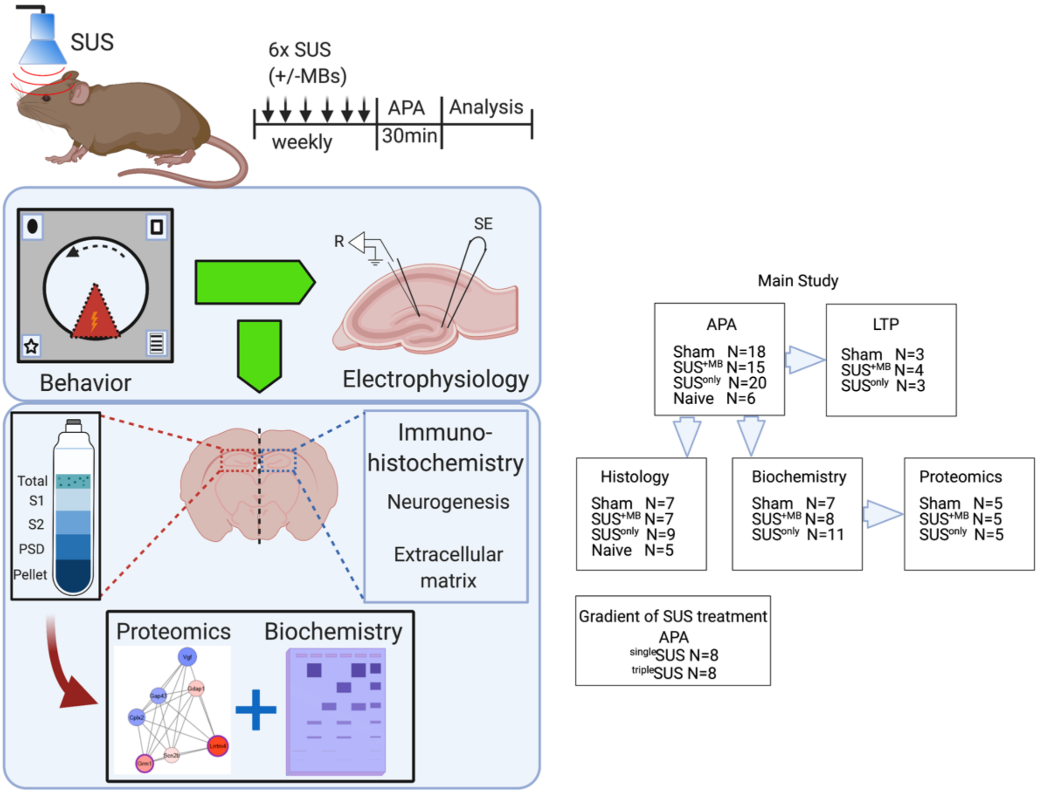

Supplementary Figure 1| Experimental design. 20 month-old animals were treated with SUS with or without microbubbles (SUS^{+MB} versus SUS^{only}) once a week for six weeks prior to being tested using the active place avoidance (APA) paradigm. A cohort of these animals was tested to determine if they exhibited long-term potentiation (LTP). The remaining animals were sacrificed by transcardial perfusion with PBS, after which one hemisphere of the brain was prepared for biochemical analysis in the form of Western blots or proteomic analysis, with the remaining hemisphere being used for immunohistochemistry.

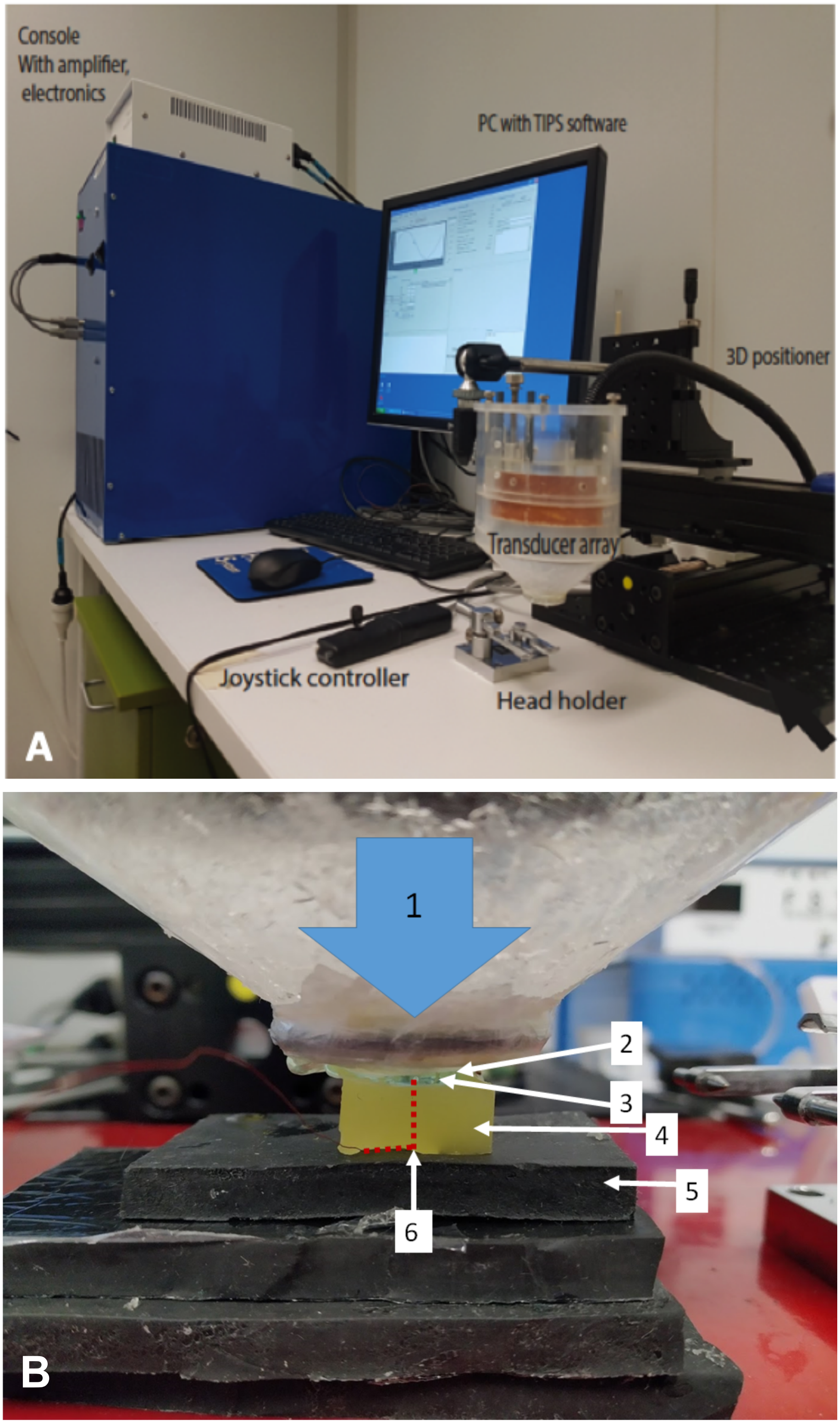

Supplementary Figure 2| The scanning ultrasound equipment is programmable to accurately deliver treatment. (**A**) The Therapy Imaging Probe System (TIPS) from Philips Research was used to deliver scanning ultrasound (SUS) to ketamine-anesthetized mice. The anesthetized animals were immobilized in the head holder such that the ultrasound could be accurately and reproducibly delivered. The transducer position was controlled by a 3D positioner to ensure accuracy. (**B**) The tissue phantom thermal test set-up was as follows. 1: Ultrasound path, 2: acoustic coupling gel, 3: skull piece, 4: gray matter mimetic acoustic gel, 5: acoustic absorber, and 6: bare-wire thermocouple.

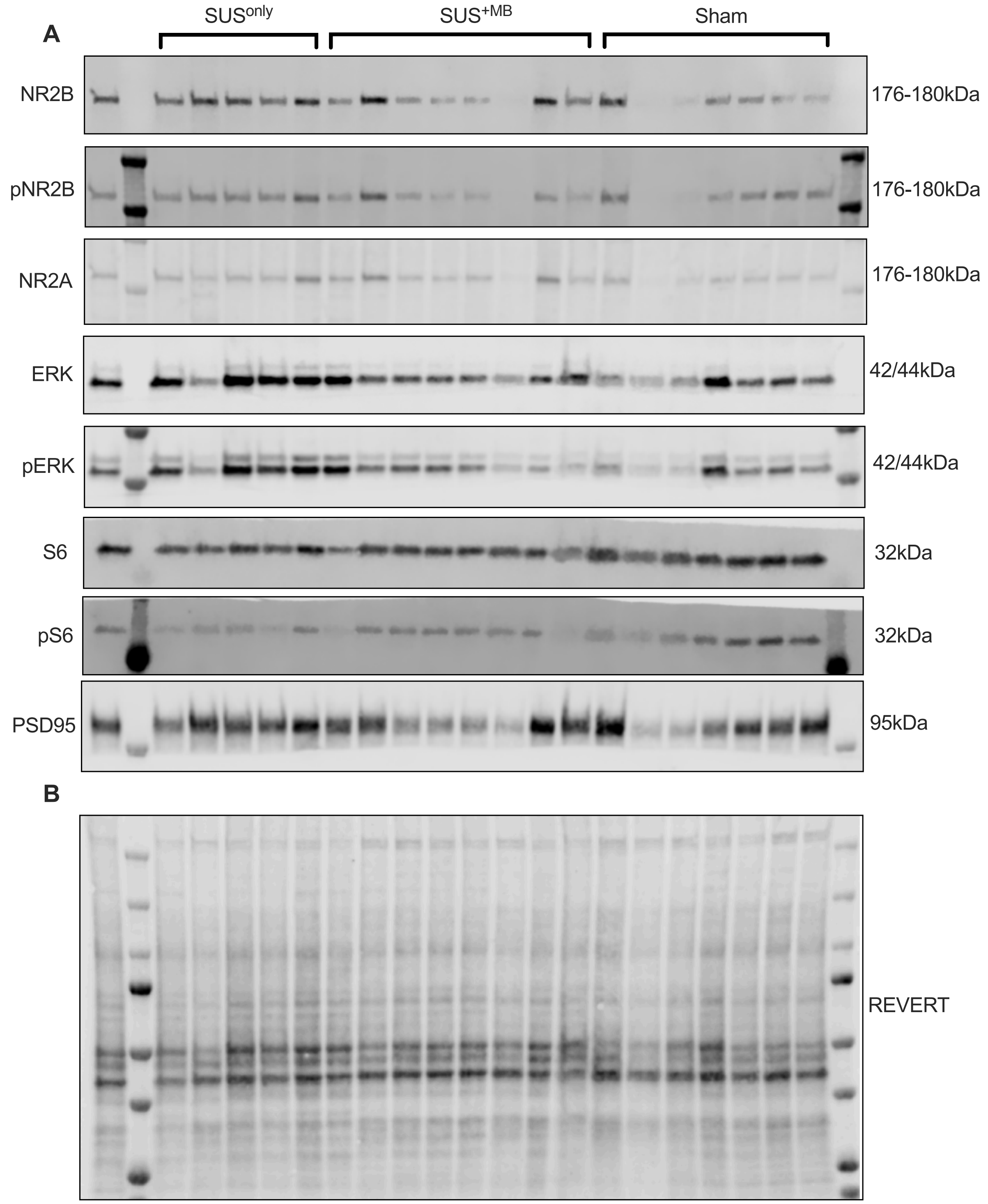

Supplementary Figure 3 | Representative western blot analysis of the hippocampal postsynaptic (PSD) protein fraction following SUS treatment. (**A**) Western blots showing specific hippocampal proteins from the PSD fraction. (**B**) Entire western blot of the PSD fraction labeled with REVERT to allow standardization of proteins for analysis.

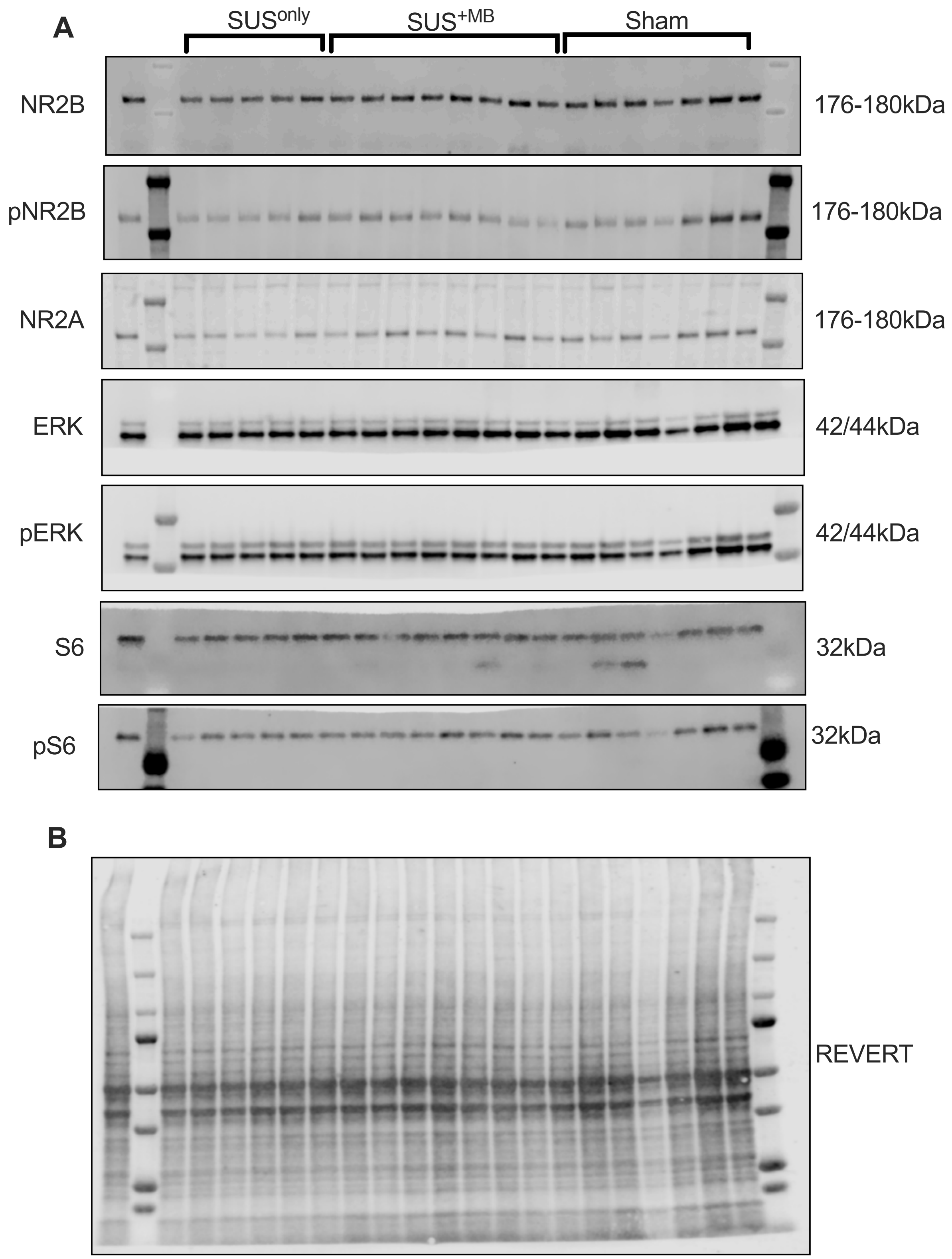

Supplementary Figure 4 | Representative western blot analysis of the hippocampal total protein fraction following SUS treatment. (**A**) Western blots showing specific hippocampal proteins from the total protein fraction. (**B**) Entire western blot of total protein fraction labeled with REVERT to allow standardization of proteins for analysis.

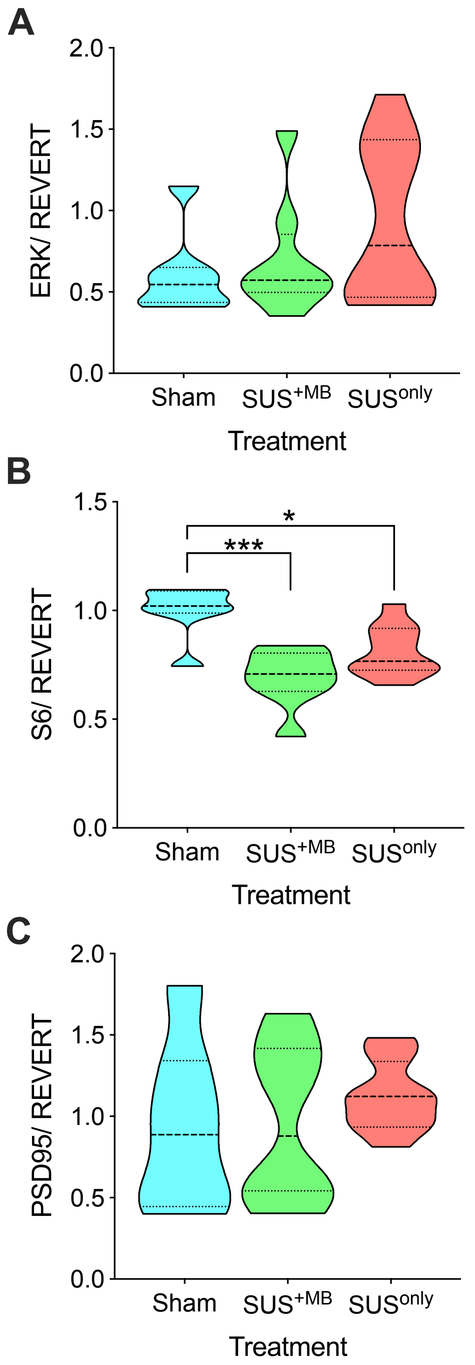

Supplementary Figure 5 | SUS treatment alters the levels of hippocampal proteins in the postsynaptic density fraction. Quantification of data in supplementary figure 4. (**A**) There was no difference in ERK levels in any of the three groups investigated. (**B**) S6 was increased in the postsynaptic fractions from both SUS^{+MB} and SUS^{only} mice compared to the sham group (one-way ANOVA [F(2,23)= 12.10, p=0.0003], with Bonferroni post-hoc analysis *p<0.05, ***p<0.001). (**C**) PSD95 was not altered in either of the SUS groups compared to the sham controls (one-way ANOVA, with Bonferroni post-hoc analysis).

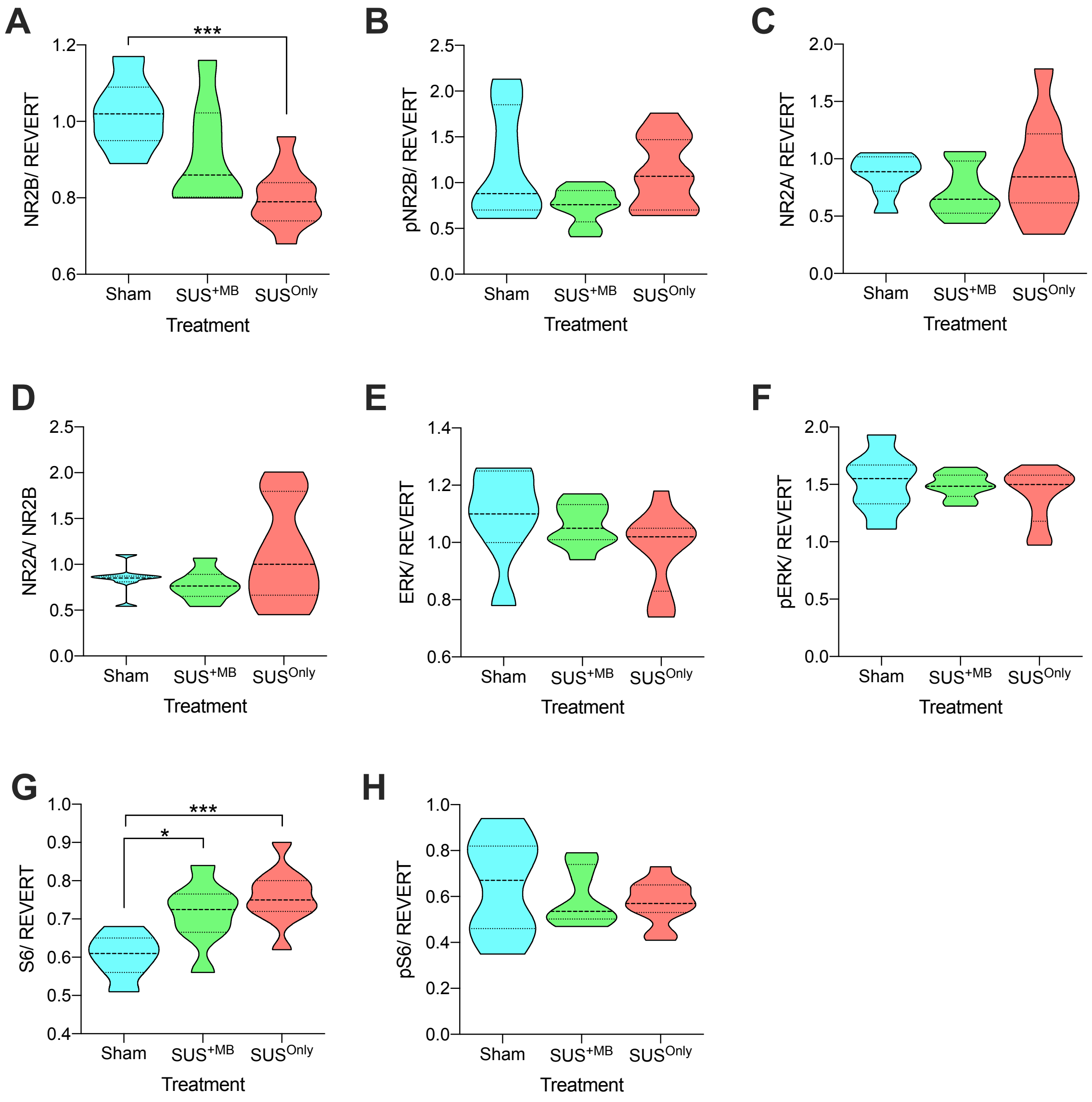

Supplementary Figure 6 | SUS treatment alters the levels of hippocampal proteins in the total protein fraction. Quantification of data in supplementary figure 4. (**A**) NR2B was decreased in the total hippocampal fraction from SUS^{only} mice compared to sham animals (one-way ANOVA [F(2,23)= 9.889, p=0.0008], with Bonferroni post-hoc analysis, ***p<0.001). There was no change in pNR2B (**B**), NR2A (**C**), or the NR2A/NR2B ratio (**D**) in the total hippocampal fractions following SUS treatment. (**E**) ERK and (**F**) pERK were not altered following SUS treatment. (**G**) S6 was increased following both SUS^{+MB} and SUS^{only} treatments (one-way ANOVA [F(2,23)= 9.825, p=0.0003], with Bonferroni post-hoc analysis *p<0.05, ***p<0.001). (**H**) There was no change in pS6 levels following SUS treatment.

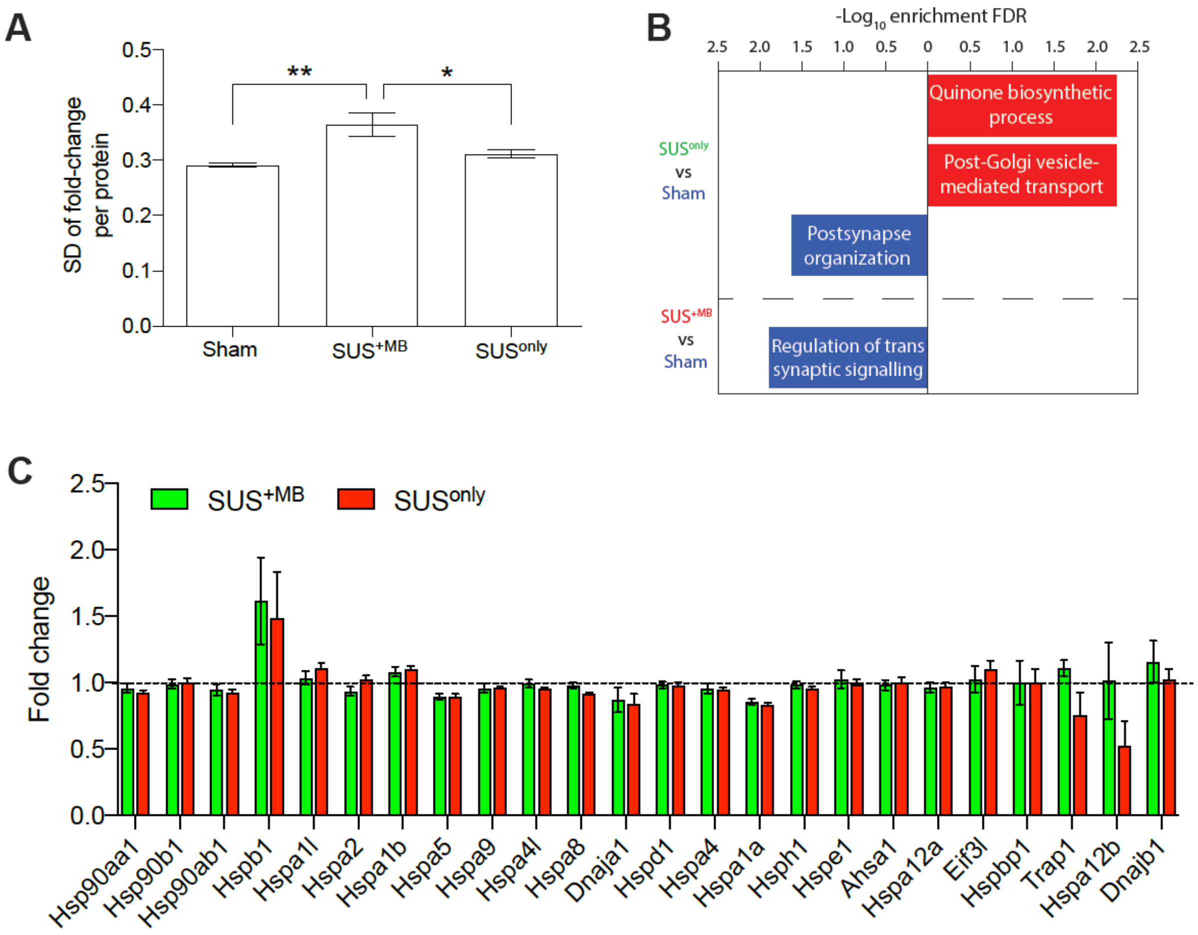

Supplementary Figure 7 | SUS treatment alters the proteomic profile of hippocampal proteins. (**A**) SUS^{+MB} produced a higher level of variation in protein fold changes relative to sham animals following treatment (one-way ANOVA [F(1.117,3588)= 8.988, p=0.0019], with Bonferroni post-hoc analysis, *p<0.05 and **p<0.01). **(B)** SUS^{only} animals showed significant fold changes in proteins associated with key pathways including postsynaptic organization, intracellular sorting and mitochondrial function. (**C**) There was no difference in heat shock proteins following SUS treatment. The dotted line represents the Sham treated average set at 1.0.

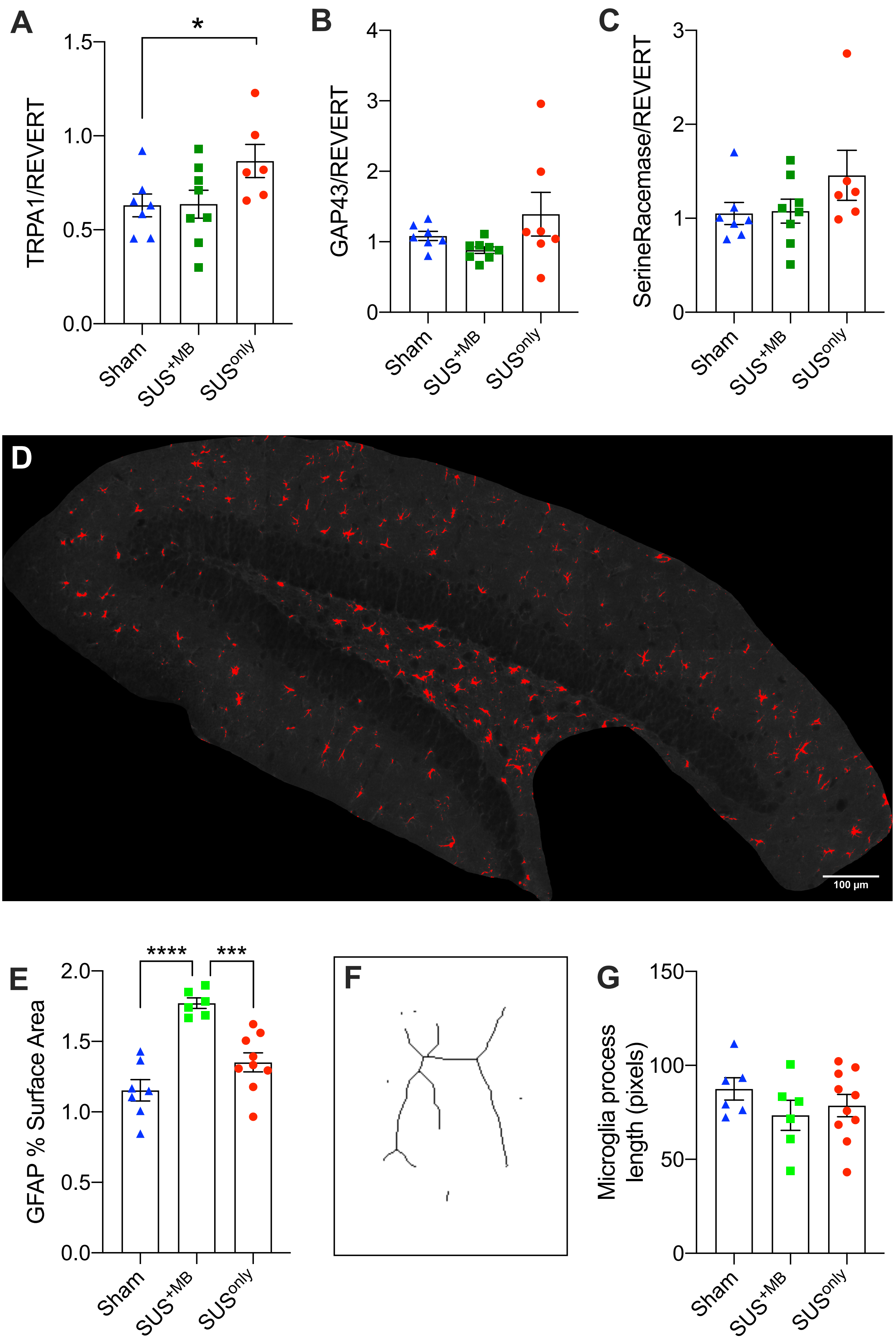
A
*
1.5
1.0
0.5
0.0
TRPA1/REVERT
Sham
SUS+MB
SUSonly
B
4
3
2
1
0
GAP43/REVERT
Sham
SUS+MB
SUSonly
C
3
2
1
0
SerineRacemase/REVERT
Sham
SUS+MB
SUSonly
D
100 µm
E
**** ***
2.0
1.5
1.0
0.5
0.0
GFAP % Surface Area
Sham
SUS+MB
SUSonly
F
G
150
100
50
0
Microglia process length (pixels)
Sham
SUS+MB
SUSonly

Supplementary Figure 8 | SUS treatment alters astrocytosis in SUS^{+MB} animals and increases TRPA1 protein levels in SUS^{+only} animals. (**A**) There was a significant increase in the calcium-permeable nonselective cation channel TRPA1 for SUS^{only} animals in hippocampal total protein fractions (student t-test), but no change for either GAP43 (**B**) or serine racemase (**C**). (**D**) Representative photomicrograph of the hippocampal dentate gyrus showing GFAP^{+ve} astrocytes labelled in red. (**E**) The % surface area of GFAP^{+ve} cells was calculated and showed a significant increase in the SUS^{+MB} treated mice (one-way ANOVA with Bonferroni post-hoc analysis). (**F**) Representative skeleton plot of Iba^{+ve} microglia. (G) There was no change between treatments for microglial process length. *p<0.05, ***p<0.001, ****p<0.0001.

Supplementary text

Heat considerations:

The Pennes bio-heat transfer equation (BHTE) [1] has been widely used to estimate tissue heating in response to focused ultrasound as a function of time [2,3]. In order to predict heating in the skull and brain tissue, BHTE was adopted and solved using parameters in the two types of SUS treatments of this study (SUS^{+MB} and SUS^{only}). BHTE is given as

$$\rho_t c_t \frac{\partial T}{\partial t} = k_t \nabla^2 T + W_b c_b (T_a - T) + \alpha \frac{P^2}{\rho c}, \quad (1)$$

where ρ_t is the tissue density, c_t is the specific heat of tissue, k_t is the tissue thermal conductivity, W_b is the blood perfusion rate, c_b is the specific heat of blood, T_a is the aterial temperature, T is the tissue temperature, α is the acoustic absorption coefficient, P is the pressure, ρ is the density and c is the velocity of sound.

As seen in Eq. (1), the left-hand side of the BHTE describes the heating rate, which is the temperature rise per unit time, whereas the right-hand side describes conduction, perfusion, and ultrasound absorption by the tissue in the target area, respectively. Given that we aimed to estimate the upper boundary of the temperature rise, two effects related to cooling, heat convection and perfusion, were excluded, and BHTE was simplified as follows

$$\rho_t c_t \frac{\partial T}{\partial t} = \alpha \frac{P^2}{\rho c} = \alpha \cdot I_{spta}, \quad (2)$$

whereby I_{spta} is the spatial peak temporal average intensity.

(i) Skull heating

Heating of the mouse skull for SUS treatments was estimated by replacing the absorption coefficient, density, and specific heat of tissue in Eq. (2). Because the property values of the mouse skull were not available, those of the human skull [7] were used in the calculation and the heating rate was calculated as

$$\frac{\partial T}{\partial t} = \frac{31.1\, Np/m \times 17\, kW/m^2}{1850\, kg/m^3 \times 1600\, J/kg{\cdot}°C} = 0.18\, °C/s.$$

As a result, the maximal temperature rise after 6 s sonication was estimated to be 1.1°C in the skull.

Table 1. List of skull properties and acoustic parameter values used in the estimation of skull heating by ultrasound.

Absorption coefficient	α_0	31.1 Np/m (= 2.7 dB/cm at 1 Hz)
Density	ρ_t	1850 kg/m^3
Specific heat of tissue	c_t	1600 J/kg°C
Peak pressure	P	0.7 MPa.<br><br>Note that it was assumed that skull was placed at the focus.
Acoustic intensity (spatial peak temporal average)	I_{spta}	17.0 kW/m^2 (= 1.7 W/cm^2)

(ii) Brain tissue heating in the absence of microbubbles

Using values applied in our SUS protocol and tissue properties reported in the literature (see Table 2), Eq. (2) is first solved as below

$$\frac{\partial T}{\partial t} = \frac{2.9\, Np/m \times 11\, kW/m^2}{1000\, kg/m^3 \times 4184\, J/kg{\cdot}°C} = 0.0076\, °C/s.$$

The temperature increase can then be calculated by integrating the heating rate over treatment time which was 6 s for all SUS sonications. Therefore, a maximal temperature increase of $0.0076\, °C/s \times 6\,\text{s} = 0.046\, °C$ was obtained.

Table 2. List of tissue properties and acoustic parameter values used in the estimation of brain tissue heating by SUS^{only}.

Absorption coefficient	α_0	2.9 Np/m at 1 MHz [4]
Density	ρ_t	1000 kg/m^3
Specific heat of tissue	c_t	4184 $J/kg \cdot °C$
Peak pressure	P	0.57 MPa.<br><br>Note that the peak pressure is assumed to be the identical to the peak negative pressure and that an 18% skull attenuation was applied [5]. As a result, 0.57 MPa (= 0.7 × 0.82 MPa) was obtained.
Acoustic intensity (spatial peak temporal average)	I_{spta}	11 kW/m^2 (= 1.1 W/cm^2) was calculated as below<br><br>$I_{spta} = \frac{1}{T_{PRI}} \int_0^{T_{PL}} \frac{P(t)^2}{\rho c} dt$, where T_{PL} is the pulse length, 10 ms, and T_{PRI} pulse repetition interval, 100 ms.

To validate our theoretical calculation, using parameters obtained from the literature [3] that compared the theoretical estimation to the experimental measurement using tissue-mimicking phantom and a thermocouple, Eq. (2) was solved as

$$\frac{\partial T}{\partial t} = \frac{18.4\ Np/m \times 625\ kW/m^2}{1100\ kg/m^3 \times 3300\ J/kg{\cdot}°C} = 3.17\ °C/s.$$

Integrating the calculated heating rate for 1.2 s resulted in a 3.8°C temperature rise, which is comparable to the estimated increase in Fig. 2 provided in [3].

Based on the two calculations above, we conclude that the temperature rise in brain tissue without microbubbles (SUS^{only}) would be less than 0.046°C.

(iii) Brain tissue heating in the presence of microbubbles.

For the SUS^{+MB} treatment, the temperature rise in brain tissue is higher as it is well established that the presence of microbubbles at the target accelerates heat deposition. Based on [6], it is predicted that the heating rate will be 3-6 times higher than for SUS^{only}. This translates to a maximal temperature rise of $0.0076\ °C/s\ \times 6 \times 6\ s = 0.27\ °C$.

References

[1] H. H. Pennes. Analysis of tissue and arterial blood temperatures in the resting human forearm. *J. Appl. Physiol.* **1**, 93-122 (1948).

[2] J. S. Jeong. Dual concentric-sectored HIFU transducer with phase-shifted ultrasound excitation for expanded necrotic region: A simulation study. *IEEE Tran. Ultrason. Ferroelectr. Freq. Control* **60**, 924-931 (2013).

[3] R. G. Holt, R. A. Roy. Measurements of bubble-enhanced heating from focused, MHz-frequency ultrasound in a tissue-mimicking material. *Ultrason. Med. Biol*. **27**, 1399-1412 (2001).

[4] S. A. Goss, L. A. Frizzel, F. Dunn. Ultrasonic absorption and attenuation in mammalian tissues. *Ultrason. Med. Biol*. **5**, 181-186 (1979).

[5] J. J. Choi, M. Pernot, S. A. Small, E. E. Konofagou. Noninvasive, transcranial and localized opening of the blood-brain barrier using focused ultrasound in mice. *Ultrason. Med. Biol*. **33**, 95-104 (2007).

[6] C. C. Coussios, C. H. Farny, G. Ter Haar, R. A. Roy. Role of acoustic cavitation in the delivery and monitoring of cancer treatment by high-intensity. *Int. J. Hyperthermia* **23**, 105-120 (2009).

[7] G. Pinton, J.-F. Aubry, E. Bossy, M. Muller, M. Pernot, M. Tanter. Attenuation, scattering, and absorption of ultrasound in the skull bone. *Med. Phys.* **39**, 299-307 (2012).
